# The effect of different timing of blood transfusion on oncological outcomes of patients undergoing radical cystectomy for bladder cancer: a systematic review and meta-analysis

**DOI:** 10.3389/fonc.2023.1223592

**Published:** 2023-08-30

**Authors:** Si-Yang Ma, Ye An, Jian-Xuan Sun, Meng-Yao Xu, Chen-Qian Liu, Jin-Zhou Xu, Xing-Yu Zhong, Na Zeng, Hao-Dong He, Qi-Dong Xia, Shao-Gang Wang

**Affiliations:** Department and Institute of Urology, Tongji Hospital, Tongji Medical College, Huazhong University of Science and Technology, Wuhan, China

**Keywords:** bladder cancer, radical cystectomy, blood transfusion, oncological, meta-analysis, systematic review

## Abstract

**Highlights:**

This meta-analysis and systematic review aim to analyze the association between BT and oncological outcomes of patients undergoing RC for bladder cancer, and tries to find out whether the timing of blood transfusion could also have an effect on this relationship. A total of 20 retrospective studies from online databases and other sources are identified and enrolled in this study. The results show that BT administration during RC operation or perioperative period is significantly associated with worse oncological outcomes including ACM, CSM and DR.

**Background:**

Bladder cancer is one of the most common urological malignancies. Radical cystectomy (RC) remains the main treatment for localized muscle-invasive bladder cancer (MIBC) or high-grade non-muscle-invasive bladder cancer (NMIBC). In the process of RC, the administration of blood transfusion (BT) is sometimes needed, however, it may cause transfusion-related complications or lead to worse oncological outcomes. This meta-analysis and systematic review aims to give a comprehensive insight into the association between BT and oncological outcomes of patients undergoing RC, and tries to find out whether the timing of blood transfusion could also have an impact on this association.

**Methods:**

This systematic review and meta-analysis were carried out according to the PRISMA 2020 reporting guideline. We have searched four bibliographic databases including PubMed (Medline), EMBASE, Cochrane Library, and Web of Science with no language limitation. Studies investigating the association between BT and oncological outcomes of patients undergoing RC are identified and included in this research from inception through March 20, 2023. This research calculates the pooled hazard ratios (pHR) and 95% confidence intervals (95% CI) of all-cause mortality (ACM), cancer-specific mortality (CSM) and disease recurrence (DR) using Random Effects models or Fixed Effects models. Subgroup analyses stratified by parameters such as timing of transfusion are also conducted. This meta-analysis was registered with PROSPERO, CRD42022381656.

**Results:**

A total of 20 retrospective studies from online databases and other sources are identified and enrolled in this study. Results show that blood transfusion significantly increased the risks for ACM (HR = 1.33, 95% CI: 1.23-1.44), CSM (HR = 1.25, 95% CI: 1.15 – 1.35) and DR (HR = 1.26, 95% CI: 1.15 – 1.38). However, when stratified by the timing of BT, we find that only intraoperative and perioperative transfusion significantly increased in risks for worse prognosis, while postoperative transfusion raised none of the risks of ACM (HR = 1.26, 95% CI: 0.92-1.73), CSM (HR = 1.08, 95% CI: 0.93-1.26) nor DR (HR = 1.08, 95% CI: 0.90-1.29) significantly.

**Conclusion:**

BT administration during RC operation or perioperative period is significantly associated with worse oncological outcomes including ACM, CSM and DR. Clinicians should consider carefully when deciding to administrate BT to patients undergoing RC and carry out according to current guidelines.

## 1 Introduction

As one of the most common urological malignancies, bladder cancer was estimated to account for 81,180 new cases and 17,100 deaths in the United States in 2022 (1). It can be divided into two main categories: non-muscle-invasive bladder cancer (NMIBC) and muscle-invasive bladder cancer (MIBC). NMIBC represents about 70% of organ-confined bladder cancer while MIBC takes the remaining 30% (2). Radical cystectomy (RC), which is accompanied by pelvic lymph node dissection and urinary diversion, is still the main treatment for patients presenting localized MIBC or high-grade NMIBC (3, 4). As an invasive treatment, RC often results in a high risk of significant blood loss and requirement for blood transfusion (BT) (4, 5). However, it has been demonstrated that BT might damage organ function and increase mortality (6). Increasing studies have also discovered that the administration of BT may influence clinical outcomes of various cancer surgeries, including hepatocellular carcinoma (7), colorectal cancer (8), pancreatic adenocarcinoma (9), esophageal cancer (10) and gastric cancer (11), though their results remain highly contradictory.

The underlying mechanism for the association between BT and clinical outcomes still remains unclear. One theory proposed that BT could have immunosuppressive effect on patients and change the anti-inflammatory/pro-inflammatory milieu in the recipients (12), which may bring worse clinical outcomes.

In urological field, the effect of BT has been found to be different. Andrea et al. and Jakobsen et al. showed that BT would not bring worse clinical outcomes among patients with prostate and kidney cancer (13, 14). However, whether BT has prognostic influence on patients undergoing RC for bladder cancer is still controversial (15–17). Those studies that denied this association indicate that the effect of disease characteristics (e.g., older age, higher pathological stage, lower preoperative hemoglobin level, higher BMI and greater estimated blood loss) of patients who received BT overweighed transfusion itself, resulting in worse outcomes (17). Moreover, there were also some studies classified the timing of BT into intraoperative BT, postoperative BT and perioperative BT. Most of them found out that intraoperative BT was significantly associated with worse outcomes, while the effect of postoperative BT was controversial (18–20). Intraoperative BT refers to receiving BT during surgery, postoperative BT refers to receiving BT during hospitalization after surgery, perioperative BT refers to receiving BT during surgery or afterwards hospitalization.

Although previous meta-analyses have displayed the relationship between BT and oncological outcomes after RC (21–23), they were carried out differently in methodology and might even omit a few available evidences. In this review, we aim to examine the published articles related to BT and outcomes in patients undergoing RC more comprehensively and try to investigate whether the timing of BT also plays a role in prognosis. We conduct a systematic review of the literature and meta-analysis to test for an association between BT and all-cause mortality (ACM), cancer-specific mortality (CSM) and disease recurrence (DR) in patients who received RC.

## 2 Methods

### 2.1 Selection criteria

The inclusion criteria were as follows: (1) Original studies; (2) RC for bladder cancer; (3) Comparative studies (BT vs. no BT); (4) Reporting on oncologic outcomes after RC, such as all-cause mortality (ACM), cancer-specific mortality (CSM) or disease recurrence (DR). (5) Stratified by timing of transfusion (intraoperative vs. postoperative vs. perioperative).

The exclusion criteria were as follows: (1) Studies using cell or animal models and case reports; (2) Studies without full text or lack of usable data; (3) Studies having overlapping population

### 2.2 Search strategy

**Figure 1:**
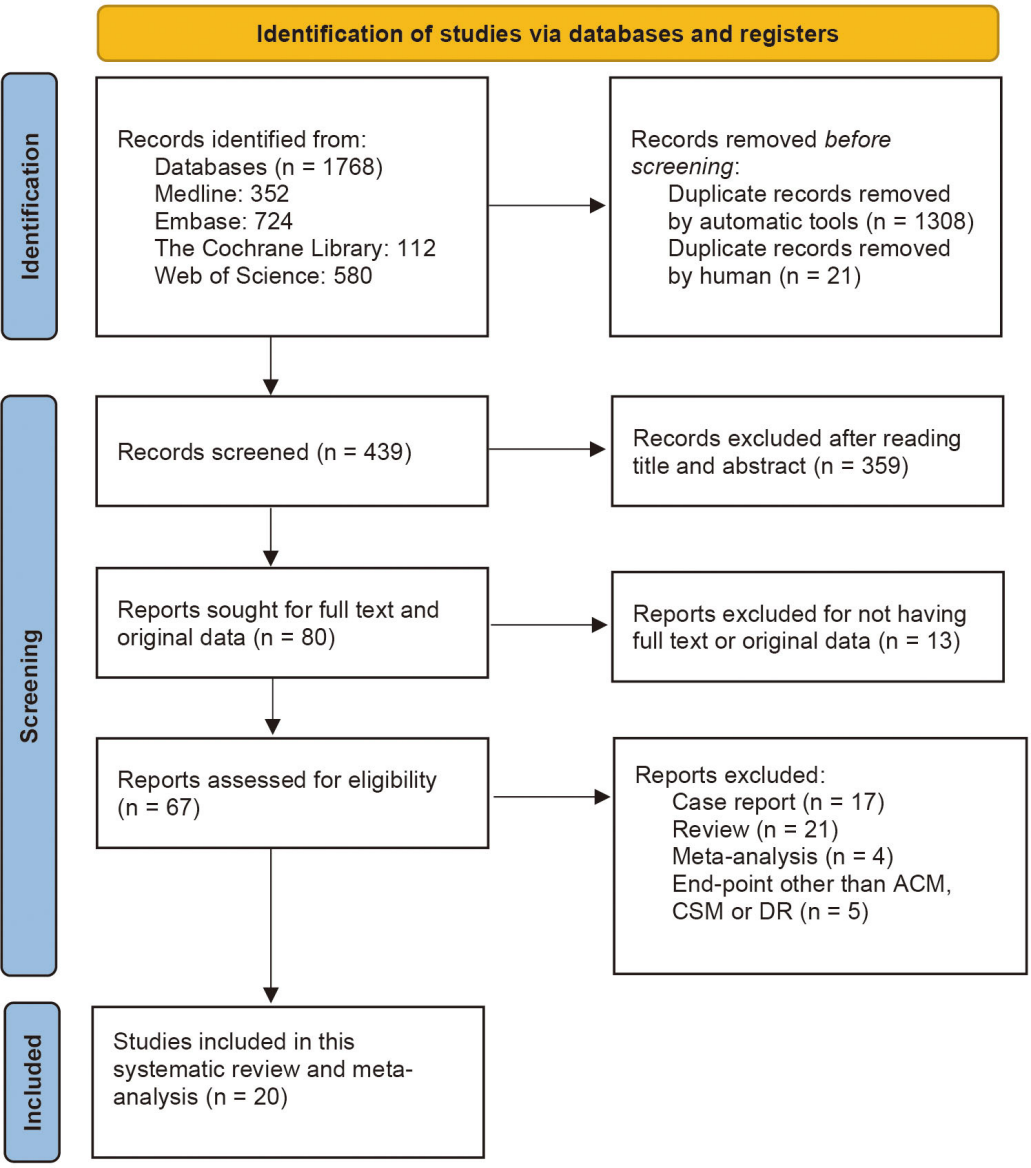
PRISMA (Preferred Reporting Items for Systematic Reviews and Meta-Analyses) flowchart for study selection for the systematic review on BT and oncological outcomes of RC. BT, blood transfusion.

This study was carried out in accordance with the Preferred Reporting Items for Systematic Reviews and Meta-analyses (PRISMA) 2020 reporting guideline (24). We have searched four bibliographic databases, including PubMed (Medline), EMBASE, Cochrane Library, and Web of Science to retrieve studies investigating the association between BT and clinical outcomes of patients receiving RC for bladder cancer from the inception to March 20, 2023. Reference lists of relevant studies, reviews and previous meta-analyses were also manually screened to identify potentially eligible studies. We used keyword terms such as “blood transfusion” and “Bladder Cancer” to search these databases. Supporting Information: **Table S1** contains the detailed search strategy for each database, including the keywords used and the number of retrieved citations per string. Two reviewers, M.S.Y and A.Y., have searched abstracts during the screening procedure and sifted them according to the search criteria. Disagreements about the inclusion or exclusion were resolved by consensus of the third author (S.J.X.). Endnote (version X9) was used to remove the duplicates and instrument the selection criteria. **Figure 1** shows the PRISMA flow chart of the literature search procedure. Before we start this systematic review and meta-analysis study, it has already been registered in PROSPERO (CRD42022381656).

### 2.3 Data extraction

Data retrieved included: (1) Bibliographic information (e.g., author’s name, year of publication and title); (2) Baseline characteristics of study participants (e.g., age, BMI, percentage of male patients and preoperative hemoglobin level); (3) Pathological characteristics of study participants (e.g., pathological stage, percentage of lymph node positive); (4) Blood loss and transfusion characteristics of study participants (e.g., estimated blood loss, volume of transfused blood and type of transfusion); (5) Outcomes of interest (e.g., all-cause mortality, cancer-specific mortality and tumor recurrence). Detailed characteristics of the articles included in this study are shown in **Table S2**.

### 2.4 Literature quality assessment

We used the Newcastle Ottawa Scale (NOS) for non-randomized to assess the quality of individual studies. The NOS assigns a maximum of nine points to eight items in three categories: selection, comparability, and outcome. Each study can be given a maximum of one point to items in the selection and outcome categories. A maximum of two points can be awarded to items in the comparability category. High quality studies referred to studies that had a score of more than six. Assessment has been carried out by two independent reviewers (M.S.Y & A.Y.). The discrepancies between reviewers have been resolved through consensus or by a third reviewer (S.J.X.). Funnel plots were used to identify publication bias.

### 2.5 Data synthesis and statistical analysis

This meta-analysis utilized Random Effects (RE) or Fixed Effects (FE) models to evaluate the relevance between hazard ratios (HR) and its 95%CI of ACM, CSM and DR and administration of BT in patients undergoing RC (25). Moreover, the pooled hazard ratio (pHR) was calculated with 95% CI using the data extracted from retrospective cohort studies to assess the effect of BT on oncological outcomes of studied population. The heterogeneity between studies was analyzed using the standard Cochrane Chi-square χ2 (Cochrane’s Q) test with a significance level of α = 0.10 and the I² test (26). The value of I² describes the percentage of variation across studies, and I² > 50% refers to considerable heterogeneity (27). The heterogeneity of included studies was also visualized by the Galbraith plots.

Subgroup analysis stratified by parameters such as continent, timing of transfusion, and type of transfusion, which could be latent reasons for heterogeneity, was performed. We also carried out meta-regression analysis instead of subgroup analysis for continuous variables such as such as age, follow-up period, percentage of male patients, BMI, preoperative hemoglobin level and percentage of different pathological stages to find out potential confounders among the studies. The publication bias of retrieved studies was analyzed by using both the Begg’s and Egger’s tests (28, 29). A funnel plot was used to determine other causes of publication bias by observing the symmetry. Finally, we did a sensitivity analysis and applied the trim and fill method to further evaluate the effect of publication bias (30). A filled forest plot was also constructed to eliminate the publication bias on pHR. This study used the R software version 4.2.0 with the “meta” package and “metagen” command for data synthesizing and statistical analysis. All the p-values were two-sided, and a p < 0.05 was considered significantly different.

## 3 Result

### 3.1 Study selection

We identified a total of 1,768 publications from online databases and other sources. After executing the inclusion and exclusion criteria in **Figure 1**, 1,748 studies were excluded, and the remaining 20 were included in this systematic review and meta-analysis. Of the 1,745 excluded articles, 1,308 duplicates were removed automatically by the Endnote application, and 21 were removed manually by reviewers. Afterwards, 359 articles were removed by reading the titles and abstracts, and 13 articles were excluded for not having full text or original data. After reading the full text, 17 case reports, 21 reviews and 4 previous meta-analyses were excluded. Another 5 articles were also removed because they had end-point events other than ACM, CSM or DR.

### 3.2 Characteristics of included studies and patients

The characteristics of included population in this study are shown in **Table S2**. We included 20 retrospective cohort studies. These studies are carried out in 8 countries and 3 continents, including Europe, America and Asia. Included articles were published between 2010 and 2023, and the individual sample sizes of each article ranged from 162 to 2895.

At a median age of 66-74 years, patients’ follow-up duration ranged from 16.1 months to 11years. The percentage of male patients varied from 63% to 82.2% and BMI ranged from 23.2 to 28.4. Only 6 studies mentioned the preoperative hemoglobin level (Hb), with a median of 11.4-13.8 g/dl. The pathological characteristic of included patients are as follows: the percentage of tumor stage greater than T2 ranged from 37% to 63.1%, and the range of percentages of patients with margin positive, high-grade tumor and lymph node positive were respectively 2-16%, 68-95.3% and 9-35%. Only a few studies mentioned the reception of chemotherapy, with a range from 3% to 75.4%. As for the characteristics of blood loss and transfusion, 19 articles comprised the timing of transfusion, among which 13 articles were about perioperative transfusion, 5 articles contained both intraoperative and postoperative transfusion and 1 article referred to intraoperative transfusion only. A total of 7 articles reported on the amount of blood transfusion, ranging from 2U to 12U. Studied patients in 12 articles received packed red blood cell (PRBC) while patients in the remaining 4 articles received leukocyte-reduced PRBC. The estimated blood loss (EBL) of included patients ranges from 400-1400 ml. The range of HR of ACM, CSM and DR reported in these articles were 0.9 to 1.77, 0.87 to 1.9 and 0.91 to 2.16 respectively.

### 3.3 Quality assessment of the included articles

The Newcastle–Ottawa scale (NOS) was applied to assess the quality of retrospective cohort studies, as shown in Supporting Information: **Table S3**. The results of NOS showed that all included articles were of high or moderate quality.

### 3.4 Association between administration of BT and oncologic outcomes after RC

#### 3.4.1 All-cause mortality

**Figure 2:**
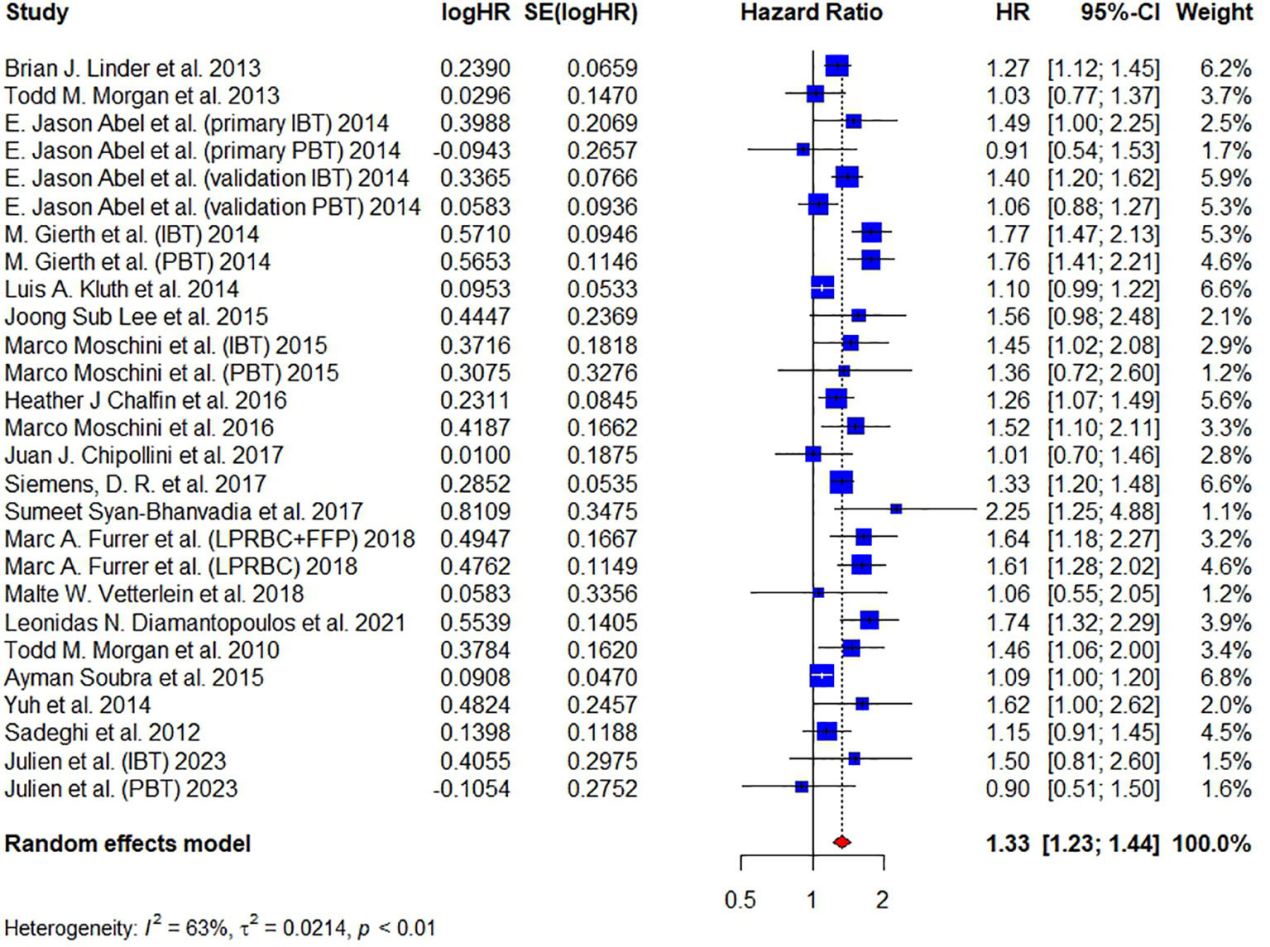
Forest plot for HR of ACM comparing between BT and no BT. Pooled HR and 95% confidence intervals of ACM, using a random-effect model. BT, blood transfusion. ACM, all-cause mortality.

Twenty articles have reported on ACM and included 18,393 patients in total. Abel et al., Gierth et al., Moschini et al. and Julien et al. divided BT into intraoperative and postoperative transfusion (18, 19, 31, 32). Abel et al. also conducted two cohorts in their article – primary and validation cohort. Pooled analysis revealed that the administration of BT was associated with the increasing of ACM, having a HR of 1.33 (95% CI: 1.23-1.44, p < 0.01), as shown in **Figure 2**. The overall heterogeneity was assessed to be considerable (I² = 63%) and RE model was used. This result is consistent with previous studies (21–23).

**Figure 3:**
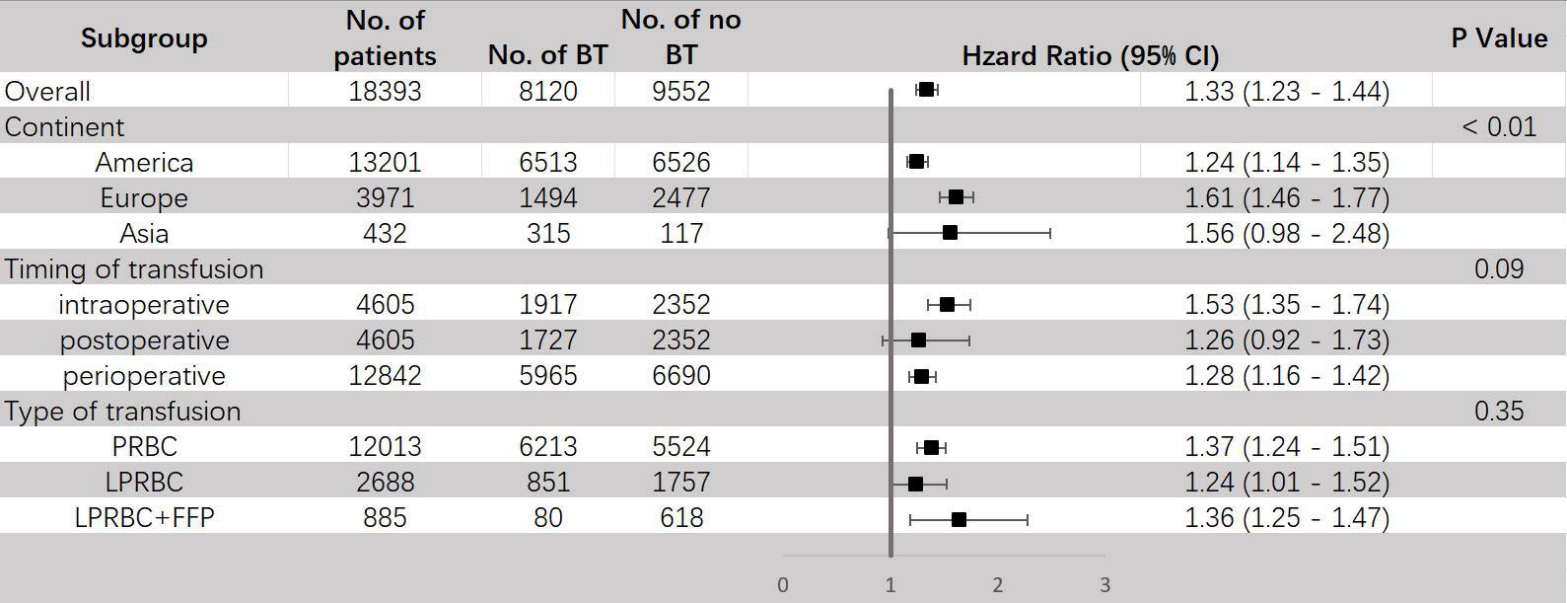
Subgroup analyses for HR of ACM comparing between BT and no BT stratified by continent, timing of transfusion and type of transfusion. BT, blood transfusion. ACM, all-cause mortality.

The results of subgroup analyses are shown in **Figure 3**. When stratified by continent, all three regions (America, Europe and Asia) showed increased mortality risks for ACM of patients receiving BT and exhibited significant difference. Furthermore, we found that the difference between these three subgroups was also significant (P < 0.01) and studied patients in Europe (HR = 1.61, 95% CI: 1.46–1.77) tended to have a higher risk of ACM than in America (HR = 1.24, 95% CI: 1.14-1.35), indicating that regions may be a potential cause of heterogeneity. As for the timing of transfusion, a significant increase in mortality risks for ACM were only observed in intraoperative (HR = 1.53, 95% CI: 1.35–1.74) and perioperative (HR = 1.28, 95% CI: 1.16–1.42) transfusion, but not in postoperative transfusion (HR = 1.26, 95% CI: 0.92-1.73). Investigating different types of transfusion revealed that both packed red blood cell transfusion (HR = 1.37, 95% CI: 1.24-1.51) and leukocyte-reduced BT (HR = 1.24, 95% CI: 1.01-1.52) significantly increased the mortality risk for ACM.

We also performed meta-regression analyses, taking into consideration the potential confounding variables, such as follow-up period, age, percentage of male patients, percentage of lymph node positive and percentage of patients in different pathological stages and found that, only the percentage of patients with high grade tumor was associated with ACM significantly (P=0.0457) (**Figure S1A**). We tried to conduct multivariable meta regression using variables with p-value less than 0.1 (**Table S4**), but failed due to lack of available data.

Besides, publication biases were tested in this meta-analysis by funnel plot and Galbraith plot. A good symmetry was not observed in the funnel plot (**Figure S2A**) and 6 studies did not locate between the dash lines in the Galbraith plot (**Figure S2B**), both of which indicated a moderate publication bias in this study. Therefore, we further conducted the trim-and-fill method to fill up the missing studies. As shown in **Figure S2C**, the funnel plot showed good symmetry after filling. The HR of filled forest plot (HR = 1.22, 95% CI: 1.11-1.33) (**Figure S2D**) was lower than the original result (**Figure 2**). Egger’s test (t = 1.77, P = 0.0886) and Begg’s test (z = 0.15, P = 0.8840) did not show significant publication bias. A sensitivity analysis was also conducted by deleting one study each time to test the stability of our results and the corresponding pooled HR of ACM did not significantly change (range from 1.32 to 1.35), indicating that our results were relatively stable (**Figure S2E**).

#### 3.4.2 Cancer-specific mortality

**Figure 4:**
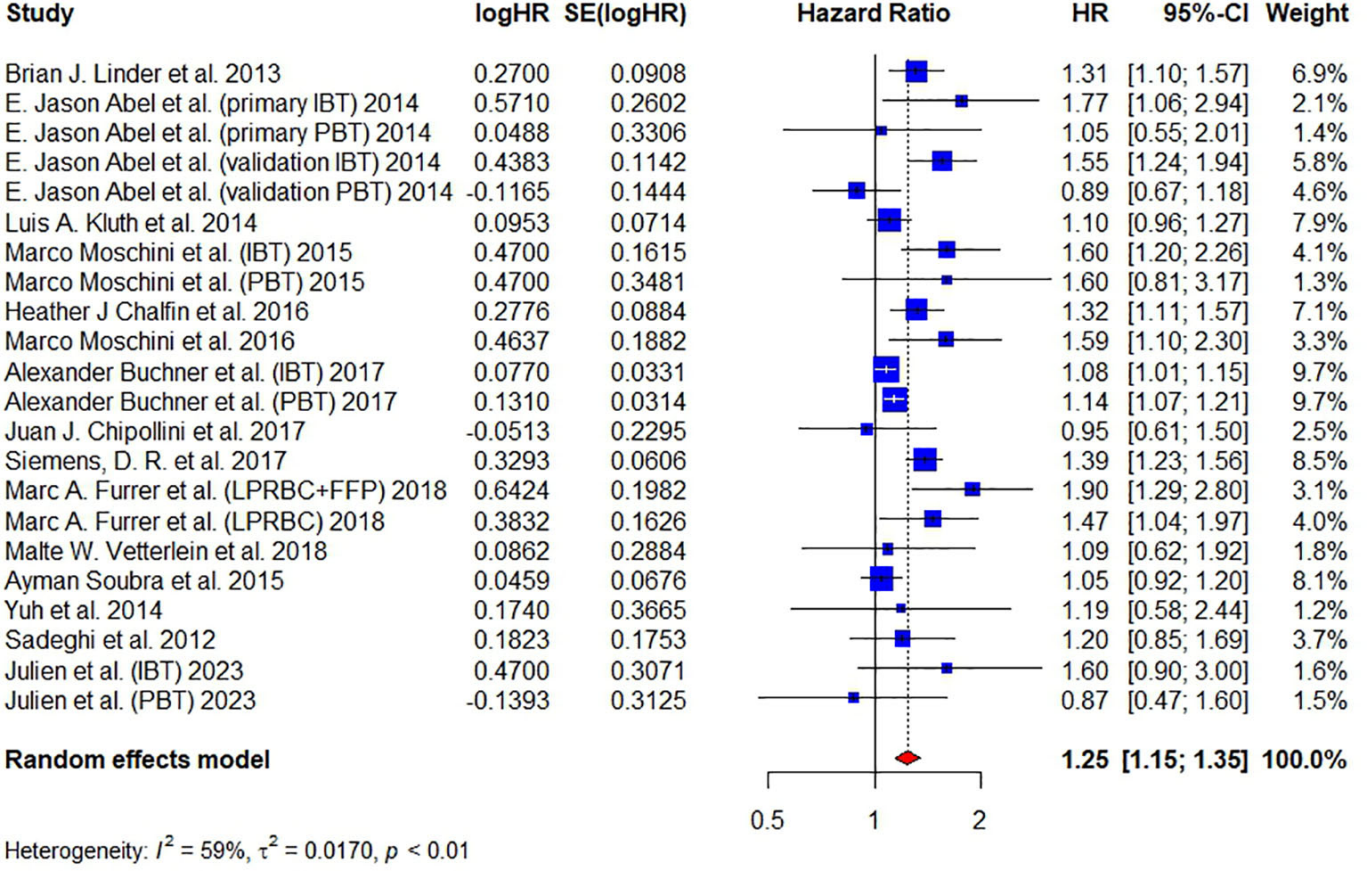
Forest plot for HR of CSM comparing between BT and no BT. Pooled HR and 95% confidence intervals of CSM, using a random-effect model. BT, blood transfusion. CSM, cancer-specific mortality.

A total of 15 articles have reported on CSM and 16,372 patients were included. Pooled analysis revealed that the administration of BT was associated with an increased CSM (HR = 1.25, 95% CI: 1.15 – 1.35, I² = 59%, RE model) (**Figure 4**).

**Figure 5:**
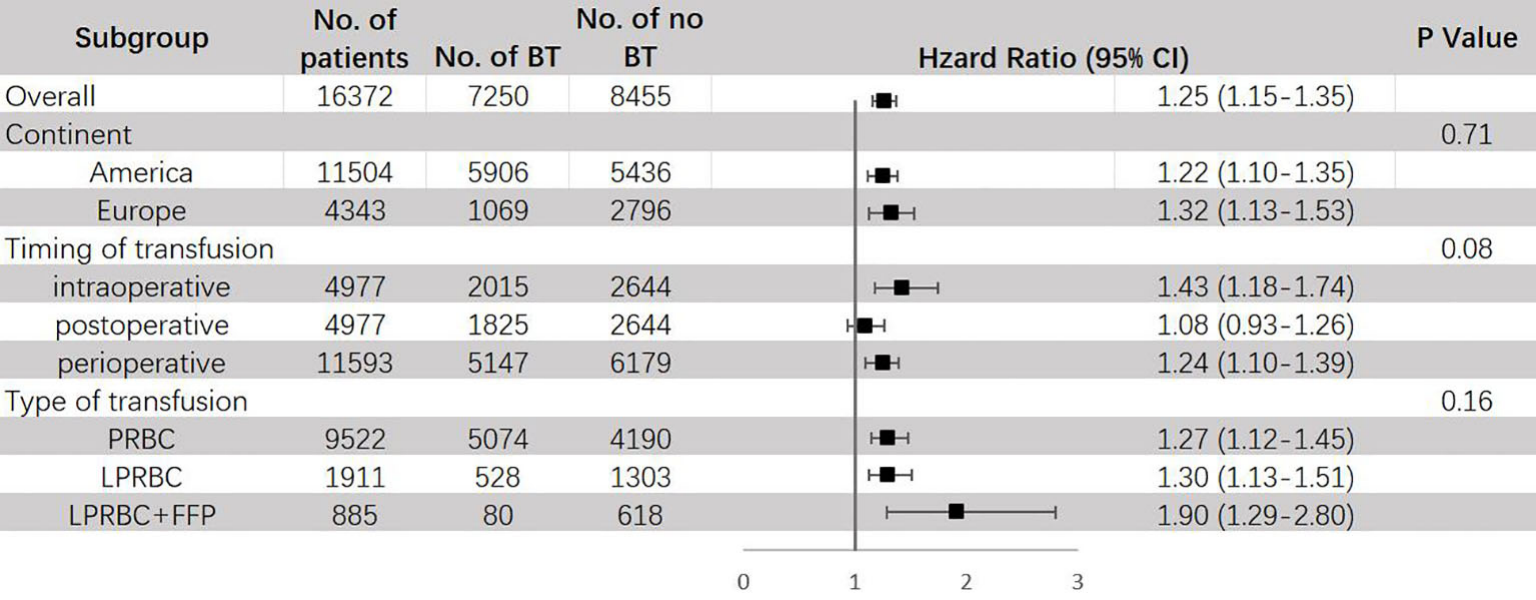
Subgroup analyses for HR of CSM comparing between BT and no BT stratified by continent, timing of transfusion and type of transfusion. BT, blood transfusion. CSM, cancer-specific mortality.

The results of subgroup analysis are shown in **Figure 5**. BT can raise the risk of CSM in both America (HR = 1.22, 95% CI: 1.10 – 1.35) and Europe (HR = 1.32, 95% CI: 1.13 – 1.53), and the difference between them was not significant. Similar as ACM, a significant increase in mortality risks for CSM were only observed in intraoperative (HR = 1.43, 95% CI: 1.18–1.74) and perioperative (HR = 1.24, 95% CI: 1.10–1.39) transfusion, but not in postoperative transfusion (HR = 1.08, 95% CI: 0.93-1.26). Both PRBC (HR = 1.27, 95% CI: 1.12 – 1.45) and leukocyte-reduced BT (HR = 1.30, 95% CI: 1.13 – 1.51) exhibited significant increase in risk for CSM, this result is inconsistent with previous study (23).

In meta-regression analysis, we discovered that for the percentages of patients with high-grade tumor (P = 0.0099) and receiving chemotherapy (P = 0.0027) were associated with CSM significantly, suggesting that these factors might be latent confounding variables, as shown in **Figure S1**. We tried to conduct multivariable meta regression using variables with p-value less than 0.1 (**Table S5**), but failed due to lack of available data.

Since good symmetry was not observed in the funnel plot and several studies were located out of the dash lines in the Galbraith plot, we continued to conduct the trim-and-fill method to fill up the missing studies. The HR of filled forest plot (HR = 1.18, 95% CI: 1.08-1.30) (**Figure S3D**) was lower than the original result (**Figure 2**). Egger’s test (t = 2.05, P = 0.0535) and Begg’s test (z = 0.03, P = 0.9775) showed that publication bias was insignificant. The sensitivity analysis showed that the corresponding pooled HR of CSM did not significantly change (range from 1.23 to 1.27), verifying the stability of our results (**Figure S3E**).

#### 3.4.3 Disease recurrence

**Figure 6:**
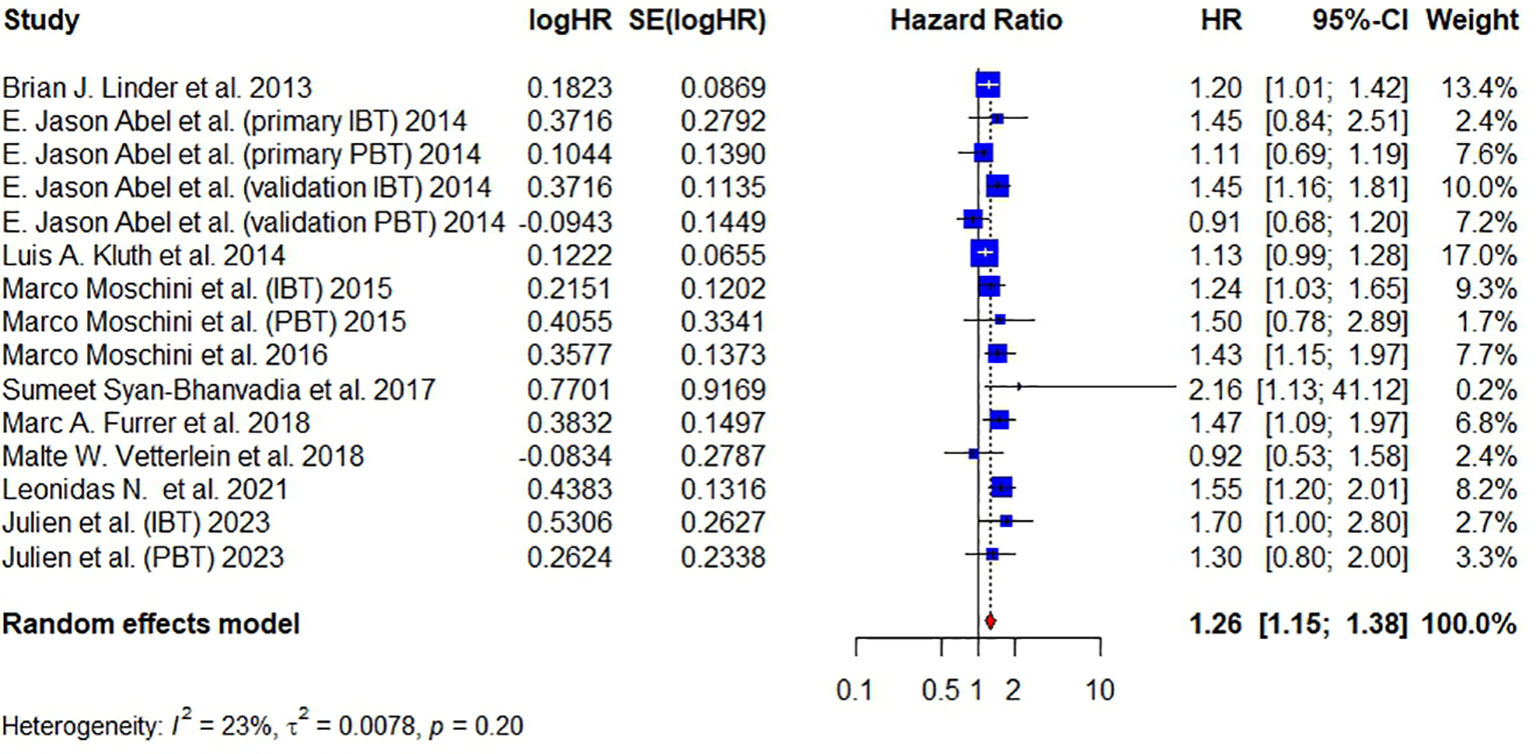
Forest plot for HR of DR comparing between BT and no BT. Pooled HR and 95% confidence intervals of DR, using a random-effect model. BT, blood transfusion. DR, disease recurrence.

Ten articles have reported on DR and a total of 11,626 patients were studied. Pooled analysis revealed that the administration of BT was associated with a significantly higher risk of disease recurrence (DR) (HR = 1.26, 95% CI: 1.15 – 1.38, I² = 23%, FE model) (**Figure 6**).

**Figure 7:**
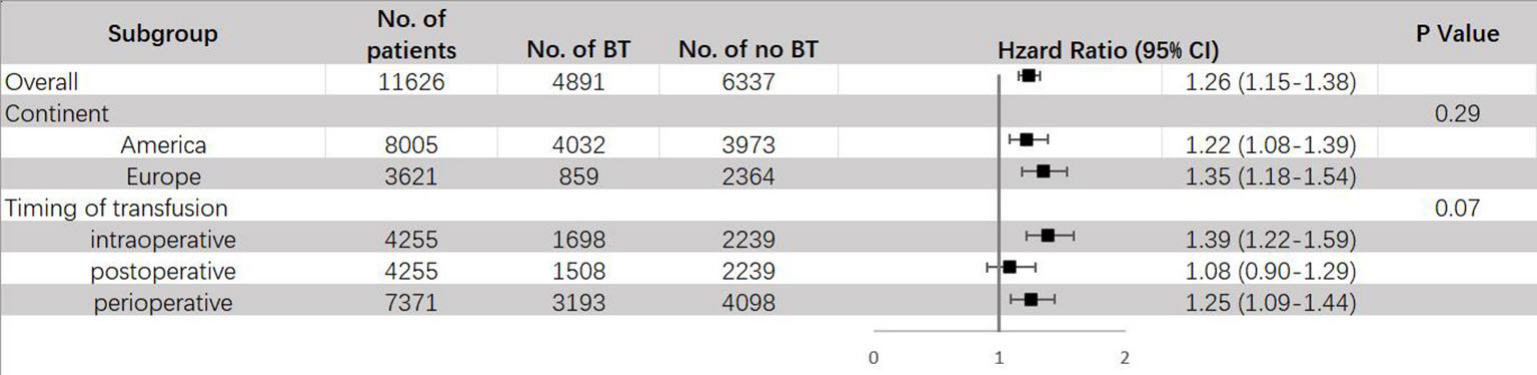
Subgroup analyses for HR of DR comparing between BT and no BT stratified by continent, timing of transfusion and type of transfusion. BT, blood transfusion. DR, disease recurrence.

The results of subgroup analysis are shown in **Figure 7**. When stratified by different regions, it was found that BT can raise the risk of DR in both America (HR = 1.22, 95% CI: 1.08 – 1.39) and Europe (HR = 1.35, 95% CI: 1.18 – 1.54). Same as ACM and CSM, a significant increase of DR risks was only observed in intraoperative (HR = 1.39, 95% CI: 1.22–1.59) and perioperative (HR = 1.25, 95% CI: 1.09–1.44) transfusion, but not in postoperative transfusion (HR = 1.08, 95% CI: 0.90-1.29). Since all articles that had data about RFS used packed red blood cell for transfusion, no subgroup analysis stratified by type of transfusion was conducted.

In meta-regression analysis, we found that publication year might be a potential confounding factor, since it was shown to associate with DR significantly (P = 0.0226) (**Figure S1E**). We tried to conduct multivariable meta regression using variables with p-value less than 0.1, but failed due to lack of available data (**Table S6**).

A relatively good symmetry was observed in the funnel plot (**Figure S4A**) and only one study was located out of the dash lines in the Galbraith plot (**Figure S4B**), suggesting that the publication bias was low. Both Egger’s test (t = 1,30, P = 0.2160) and Begg’s test (z = 0.54, P = 0.5862) did not show a significant publication bias. The sensitivity analysis showed that the corresponding pooled HR of RFS did not significantly change (range from 1.23 to 1.29), proving the stability of our results (**Figure S4C**).

## 4 Discussion

Although three meta-analyses studying the association between BT and oncological outcomes of patients undergoing RC have already been published, we found that they all had some shortages. Wang et al. and Cata et. al’ s studies were carried out in 2015 and 2016 and failed to include newly published articles, suggesting the need for an update. Moreover, they didn’t conduct subgroup analysis for parameters such as the timing of BT, perhaps due to the lack of adequate data (21, 22). Uysal et al. didn’t analyze other potential confounding factors that might influence the results such as sex and pathological stages and missed some eligible studies (23). Disadvantages mentioned above and the methodological inconsistencies between previous studies inspired us to conduct a more comprehensive meta-analysis and systematic review in the hope of helping us understand this association better and giving possible instructions to clinical surgeons.

This study combined available data in 20 published articles and concluded that the administration of BT was associated with significant increases in risks of all-cause mortality (HR = 1.33, 95% CI: 1.23-1.44), cancer-specific mortality (HR = 1.25, 95% CI: 1.15 – 1.35) and disease recurrence (HR = 1.26, 95% CI: 1.15 – 1.38). However, when stratified by different timing of BT, we found that only intraoperative and perioperative transfusion exhibited significant increase in risks for worse prognosis, while postoperative transfusion raised none of the risks of ACM (HR = 1.26, 95% CI: 0.92-1.73), CSM (HR = 1.08, 95% CI: 0.93-1.26) nor DR (HR = 1.08, 95% CI: 0.90-1.29) significantly, indicating that BT after RC surgery was not an independent predictor of clinical outcomes and only transfusion during surgery or perioperative period could lead to worse outcomes. Subgroup analyses also revealed that patients receiving BT in Europe tend to have a higher risk for worse outcomes, especially for ACM, with a p-value of difference between subgroups less than 0.01, which means that studies carried out in different continents might be a reason for heterogeneity. This might be caused by the racial differences between America and Europe.

Studies that denied the association between BT and oncological outcomes believed that the baseline or pathological characteristics of patients receiving BT, rather than BT itself, had more significant effects on relative clinical outcomes (17, 33–35). Although data included in this study were almost from multivariable cox regression models, we still conducted meta-regression analysis to try to find out other confounding factors. Our results showed that the percentage of patients with high-grade tumor was significantly associated with lower risk of ACM (P = 0.0457) and CSM (P = 0.0099). We also found that chemotherapy (P = 0.0027) significantly contributed to decreased CSM (**Figure S1**). These results could be interpreted as patients with lower tumor grades and no receipt of chemotherapy might have higher risks for unsatisfying clinical outcomes after receiving BT. Finally, we had to acknowledge that the number of available articles for our meta-regression analyses was limited, therefore, these results should be interpreted with caution.

The mechanism by which BT can influence oncological outcomes have not been constructed soundly. The existing main theories proposed that BT has an immunosuppressive effect on the recipients. For example, Vamvakas et al. found that BT could undermine the activity of monocytes and cytotoxic cells, stimulate the release of immunosuppressive prostaglandins, and increase T-cell suppressor activity, resulting in the dysfunction of immune system (36), and Blumberg et al. demonstrated that the numerous antigens carried by transfused blood cells could also lead to anergy and the disorder of immune system (37). Other studies also found that the infusion of growth factors, such as VEGF (vascular endothelial growth factor) and the host system reacting to transfused donor microparticles could promote the growth and metastasis of tumor cells (38). A further mechanism on ACM and CSM could also be represented by the presence of kidney injury proteinuria (39). All these factors might lead to a worse oncological outcome. Moreover, there are also many transfusion related complications such as acute lung injury, infections and hemolytic reactions due to ABO mismatch (40), making clinicians have to take these disadvantages of BT into account carefully.

Measures trying to reduce the adverse reactions of BT have been proposed. Considering the mechanism by which BT causes immunosuppression, Lannan et al. pointed out that leukocyte-reduced BT might had the potential to reduce the tumor promoting effects, since most white blood cells, especially neutrophiles, which can facilitate the immunosuppression effect are removed (41). Although, several studies had investigated the administration of leukocyte-reduced BT on patients undergoing RC, their results remained contradictory. Todd et al. and Chipollini et al. found that leukocyte-reduced BT was not associated with the risk of ACM significantly (16, 34), while Chalfin et al. and Furrer et al. showed the significance (42, 43). Our results of subgroup analysis stratified by different types of BT showed that leukocyte-reduced BT did not associate with increased risks of ACM (HR = 1.24, 95%CI: 1.01 – 1.52) nor CSM (HR = 1.30, 95%CI: 1.13 – 1.51) significantly, which was inconsistent with previous study (23), indicating that the efficacy of leukocyte-reduced BT still needs more evidences to verify. These findings should be interpreted with caution due to the limited number of studies included in this subgroup analysis. Another strategy is restrictive BT. Sumeet et al. have found that restrictive BT was safe for patients undergoing RC (44). By implementing this strategy, clinicians can reach a better balance between providing benefits for patients while avoiding risks of transfusion and reduce unnecessary BT (45).

Strategies that can reduce blood loss or minimize BT administration can also benefit patients undergoing RC. Henry et al. mentioned that implementing anti-fibrinolytic can reduce the need for BT (46). Crescenti et al. have also found that intraoperative use of tranexamic acid could reduce transfusion rate in radical prostatectomy (47). Preoperative Hb level is one of the main parameters considered by surgeons when deciding whether to administrate BT or not, thus by increasing preoperative Hb level or improving preoperative anemia status might also reduce the need for BT (48). Furthermore, with the development of surgical techniques such as laparoscopy-assisted RC or robot assistance may help to reduce estimated blood loss during RC, thus decreasing the need for BT (49).

This study has several limitations to be acknowledged. First, all of the studies enrolled were retrospective cohorts rather than randomized controlled trials (RCT), which indicated the existence of selection bias. However, RCT was not viable from ethical perspective, since we could not withdraw the life-saving transfusion from critically ill patients. Second, the sample sizes of included articles varied widely (from 162 to 2895), which means that the statistical weight of each study was quite different, causing bias in varying degrees. Third, the definition between endpoint events was inconsistent between included studies. For example, when analyzing the effect on disease recurrence, some studies used local or distant tumor recurrence (50), while others used survival related data such as recurrence free survival (44).

## 5 Conclusions

BT administration during RC operation or perioperative period is significantly associated with worse oncological outcomes including ACM, CSM and DR. Factors such as sex, race and pathological stages may also contribute to this association. Clinicians should consider carefully before BT administration to reduce inappropriate BT and carry out BT according to current guidelines. The efficacy of strategies that can reduce the adverse reactions or necessities of BT, such as leukocyte-reduced BT, restrictive BT or application of laparoscopy needs more evidence to be proved and novel measures to minimize BT still need more efforts to be discovered.

## Data availability statement

The original contributions presented in the study are included in the article/**Supplementary Material**. Further inquiries can be directed to the corresponding authors.

## Author contributions

S-YM, YA, J-XS, Q-DX and S-GW contributed to the developing the main research questions, carrying out the literature search, collecting the included studies’ information, and describing the results. S-YM, YA and J-XS performed the meta-analysis and wrote the first draft of the manuscript. X-YZ and J-ZX contributed to data synthesis. NZ and H-DH contributed to data extraction and revised the manuscript. C-QL and M-YX contributed to literature research, data extraction and revised the manuscript. All authors contributed to the article and approved the submitted version. S-YM, YA, J-XS, Q-DX and S-GW contributed equally to this study.
