# Supplementary material for: The effect of different timing of blood transfusion on oncological outcomes of patients undergoing radical cystectomy for bladder cancer: a systematic review and meta-analysis
Source: Front Oncol. 2023 Aug 30;13:1223592. doi: 10.3389/fonc.2023.1223592 (PMC10499617; doi:10.3389/fonc.2023.1223592)
Supplement: Supplementary file 1 [file DataSheet_1.pdf]

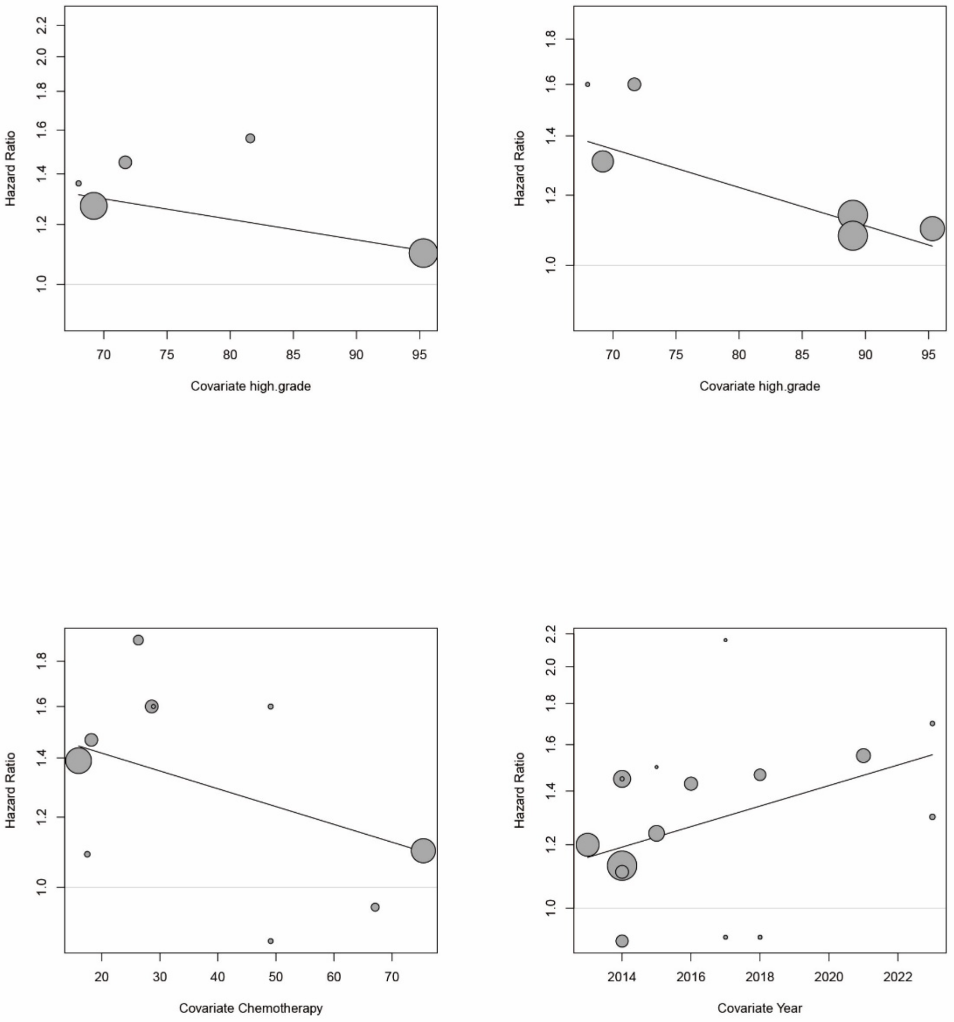

Figure S1: The bubble plots of meta-regression analyses (parameters that have shown significant difference). **(A).** The effect of percentage of patients with high-grade tumor on HR of ACM (P = 0.0457). **(B).** The effect of percentage of patients with high-grade tumor on HR of CSM (P = 0.0099). **(C).** The effect of percentage of patients receiving chemotherapy on HR of CSM (P = 0.0027). **(D).** The effect of publication year on HR of DR (P = 0.0226). ACM, all-cause mortality. CSM, cancer-specific mortality. DR, disease recurrence.

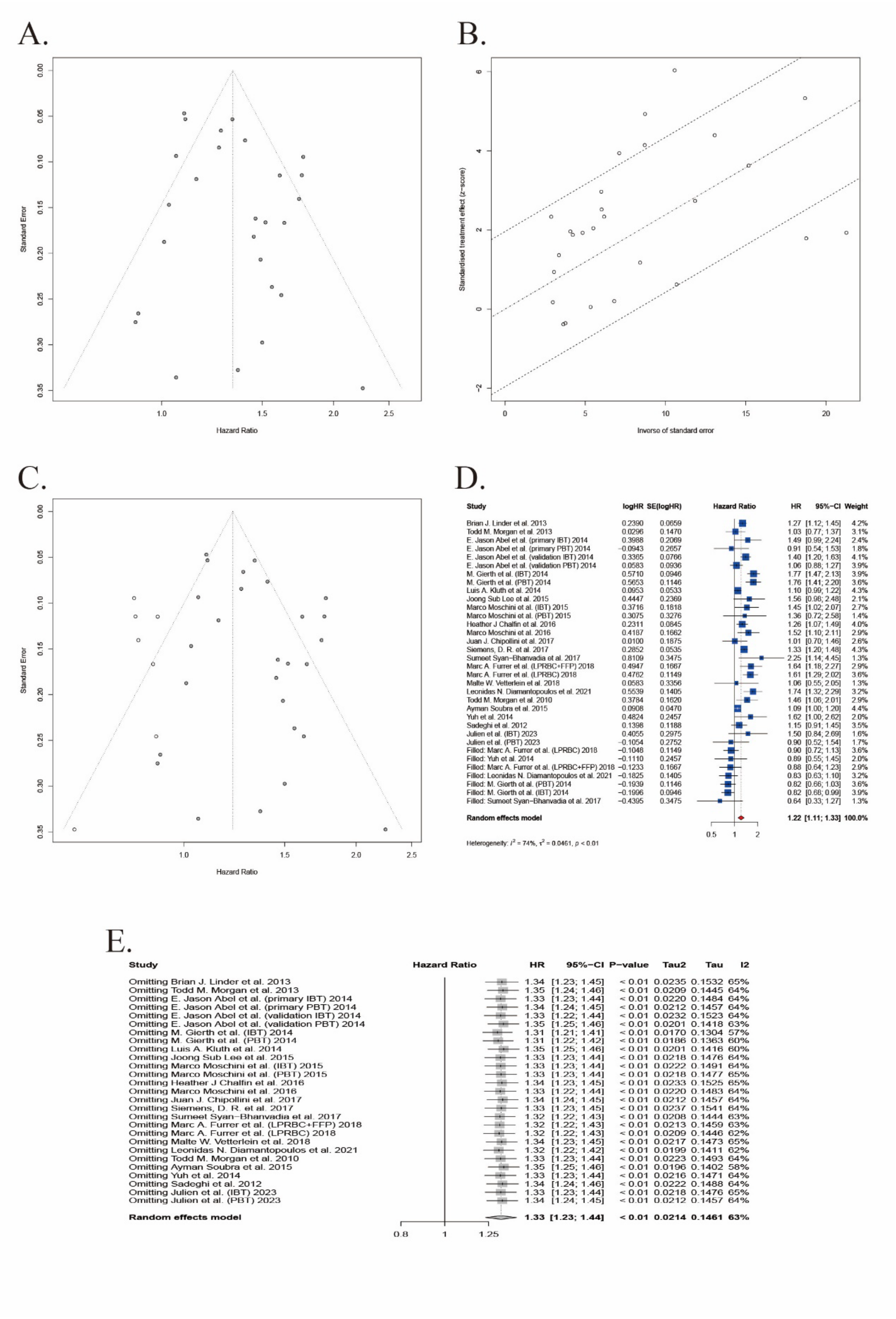

Figure S2: Diagrams about publication bias and sensitivity test of included studies in ACM: (A). The funnel plots. (B). The Galbraith plots. (C). The funnel plots after filling. (D). The forest plot after filling. (E). Sensitivity test. ACM, all-cause mortality.

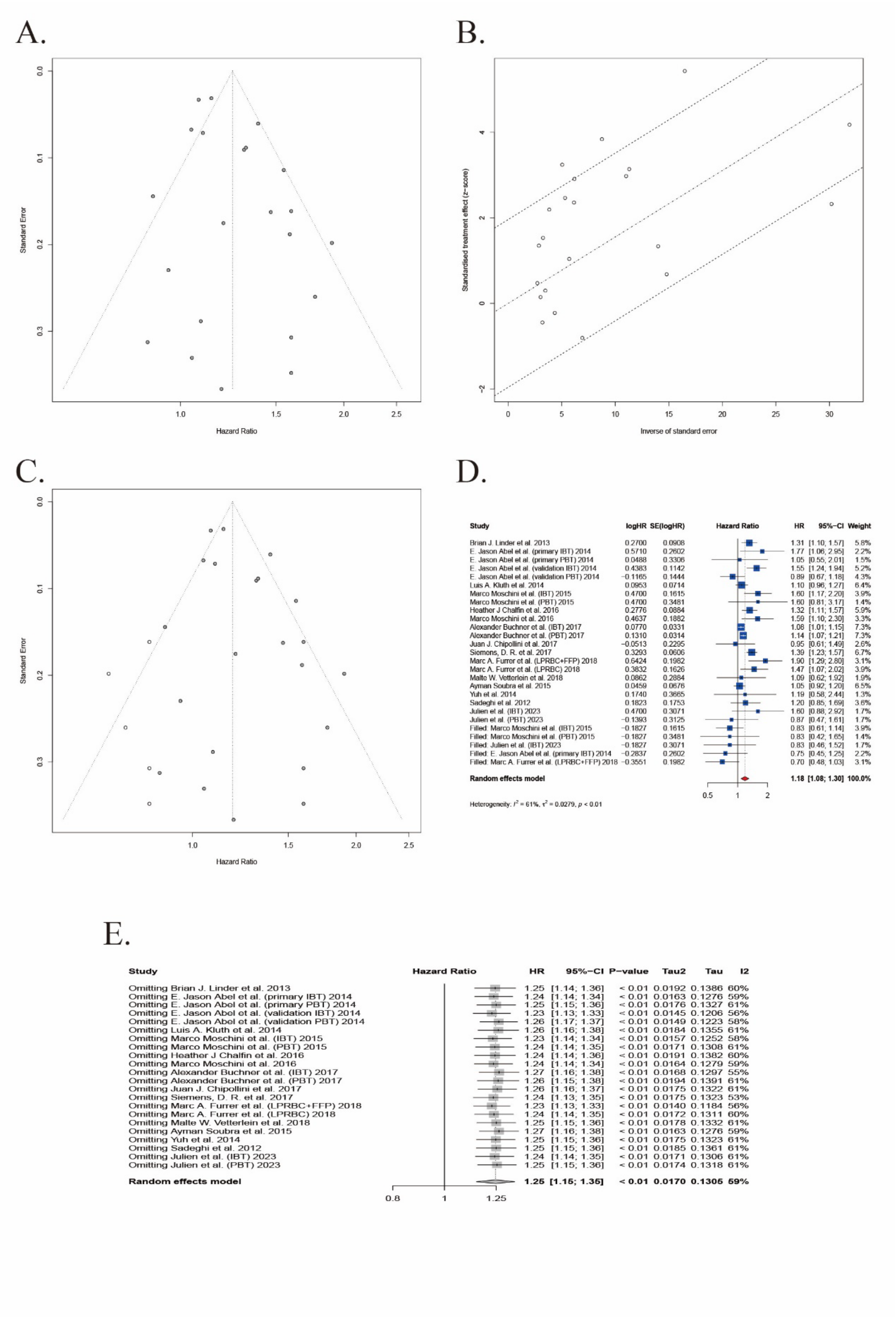

Figure S3: Diagrams about publication bias and sensitivity test of included studies in CSM: (A). The funnel plots. (B). The Galbraith plots. (C). The funnel plots after filling. (D). The forest plot after filling. (E). Sensitivity test. CSM, cancer-specific mortality.

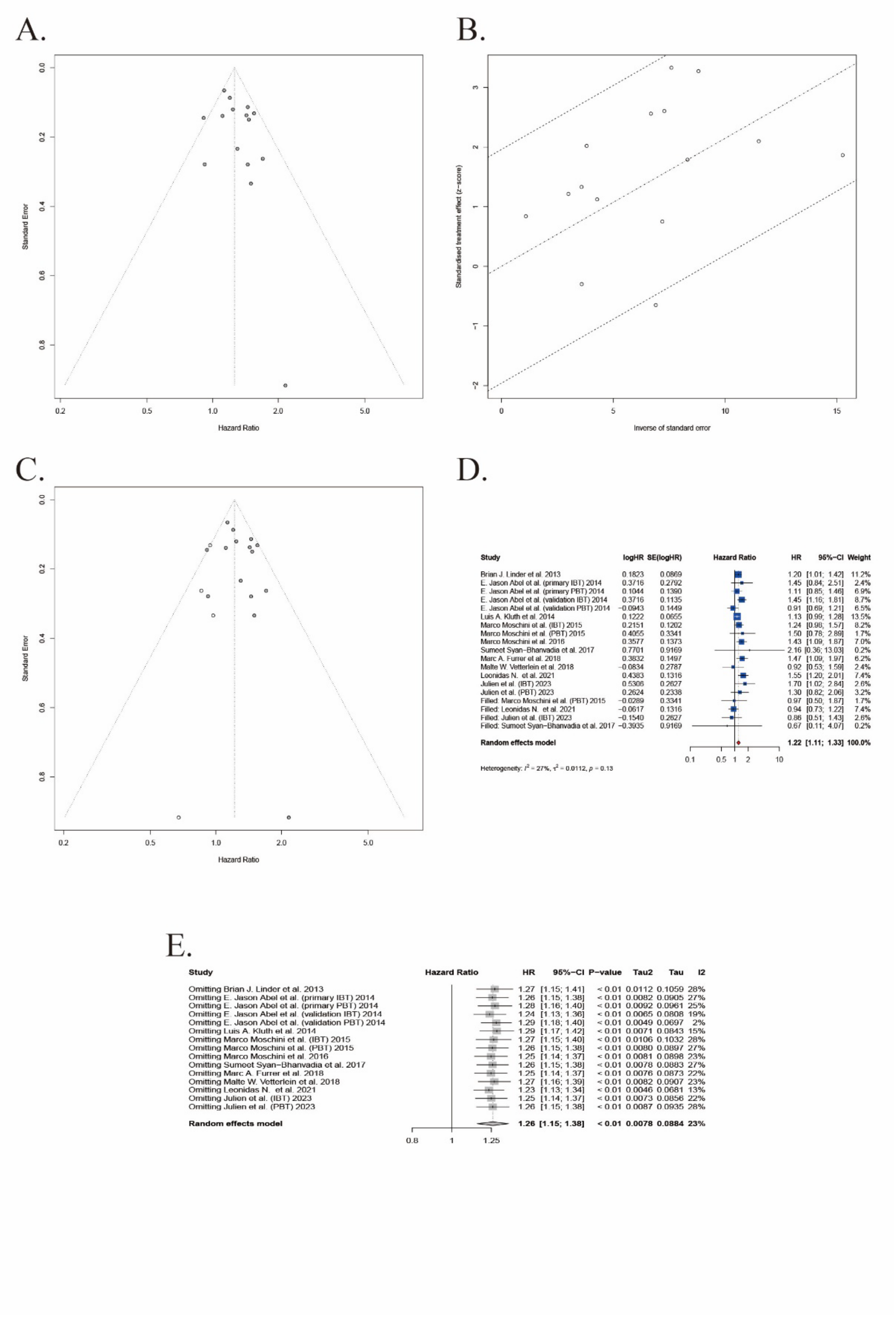

Figure S4: Diagrams about publication bias and sensitivity test of included studies in DR: (A). The funnel plots. (B). The Galbraith plots. (C). The funnel plots after filling. (D). The forest plot after filling. (E). Sensitivity test. DR, disease recurrence.
