# Supplementary material for: The effect of different timing of blood transfusion on oncological outcomes of patients undergoing radical cystectomy for bladder cancer: a systematic review and meta-analysis
Source: Front Oncol. 2023 Aug 30;13:1223592. doi: 10.3389/fonc.2023.1223592 (PMC10499617; doi:10.3389/fonc.2023.1223592)
Supplement: Supplementary file 2 [file Table_1.docx]

**Table S1A: Details of Search Strategy to Retrieve the Studies using PubMed (Medline)**

**Date of Search: 03/20/2023**

| **#** | **Search Terms** | **Hits** |
| --- | --- | --- |
| #1 | Search: "Urinary Bladder Neoplasms"[Mesh] | 61,709 |
| #2 | Search: ((((((((((((((((((Urinary Bladder Neoplasms[Title/Abstract]) OR (Neoplasm, Urinary Bladder[Title/Abstract])) OR (Urinary Bladder Neoplasm[Title/Abstract])) OR (Bladder Tumors[Title/Abstract])) OR (Bladder Tumor[Title/Abstract])) OR (Tumor, Bladder[Title/Abstract])) OR (Tumors, Bladder[Title/Abstract])) OR (Neoplasms, Bladder[Title/Abstract])) OR (Bladder Neoplasms[Title/Abstract])) OR (Bladder Neoplasm[Title/Abstract])) OR (Neoplasm, Bladder[Title/Abstract])) OR (Urinary Bladder Cancer[Title/Abstract])) OR (Cancer, Urinary Bladder[Title/Abstract])) OR (Malignant Tumor of Urinary Bladder[Title/Abstract])) OR (Cancer of the Bladder[Title/Abstract])) OR (Bladder Cancer[Title/Abstract])) OR (Bladder Cancers[Title/Abstract])) OR (Cancer, Bladder[Title/Abstract])) OR (Cancer of Bladder[Title/Abstract]) | 50,923 |
| #3 | #1 OR #2 | 75,296 |
| #4 | Search: "Blood Transfusion"[Mesh] | 91,817 |
| #5 | Search: (((Blood Transfusion[Title/Abstract]) OR (Blood Transfusions [Title/Abstract])) OR (Transfusion, Blood [Title/Abstract])) OR (Transfusions, Blood [Title/Abstract]) | 57,359 |
| #6 | #4 OR #5 | 123,906 |
| **#7** | **#3 AND #6** | **352** |

**Table S1B: Details of Search Strategy to Retrieve the Studies using Embase**

**Date of Search: 03/20/2023**

| **#** | **Search Terms** | **Hits** |
| --- | --- | --- |
| #1 | (bladder cancer or urinary bladder cancer or urine bladder cancer or vesical cancer or bladder tumor or urinary tract cancer or bladder carcinogenesis or bladder carcinoma or bladder metastasis or muscle invasive bladder cancer or non muscle invasive bladder cancer).ab,kw,ti. | 71,868 |
| #2 | (blood transfusion or [blood infusion](https://ovidsp.dc1.ovid.com/ovid-a/ovidweb.cgi?S=LACAFPOBNLACHEEEKPMJGGOIIFONAA00&Controlled+Vocabulary=thes+blood+infusion&toolSubject=blood+transfusion) or [blood replacement](https://ovidsp.dc1.ovid.com/ovid-a/ovidweb.cgi?S=LACAFPOBNLACHEEEKPMJGGOIIFONAA00&Controlled+Vocabulary=thes+blood+replacement&toolSubject=blood+transfusion) or blood retransfusion or hemotherapy or multitransfusion or polytransfusion or transfusion blood or transfusion therapy or transfusion or amnioinfusion or blood autotransfusion or blood component therapy or exchange blood transfusion or intrauterine blood transfusion).ab,kw,ti. | 176,690 |
| **#3** | **#1 AND #2** | **724** |

**Table S1C: Details of Search Strategy to Retrieve the Studies using Cochrane**

**Date of Search: 03/20/2023**

| **#** | **Search Terms** | **Hits** |
| --- | --- | --- |
| #1 | (Neoplasm, Urinary Bladder):ti,ab,kw or (Urinary Bladder Neoplasm):ti,ab,kw or (Bladder Tumors):ti,ab,kw or (Bladder Tumor):ti,ab,kw or (Tumor, Bladder):ti,ab,kw or (Tumors, Bladder):ti,ab,kw or (Neoplasms, Bladder):ti,ab,kw or (Bladder Neoplasms):ti,ab,kw or (Bladder Neoplasm):ti,ab,kw or (Neoplasm, Bladder):ti,ab,kw or (Urinary Bladder Cancer):ti,ab,kw or (Cancer, Urinary Bladder):ti,ab,kw or (Malignant Tumor of Urinary Bladder):ti,ab,kw or (Cancer of the Bladder):ti,ab,kw or (Bladder Cancer):ti,ab,kw or (Bladder Cancers):ti,ab,kw or (Cancer, Bladder):ti,ab,kw or (Cancer of Bladder):ti,ab,kw | 6,174 |
| #2 | (Blood Transfusion):ti,ab,kw or (Blood Transfusions):ti,ab,kw or (Transfusion, Blood):ti,ab,kw or (Transfusions, Blood):ti,ab,kw | 15,808 |
| **#3** | **#1 AND #2** | **112** |

**Table S1D: Details of Search Strategy to Retrieve the Studies using Web of Science**

**Date of Search: 03/20/2023**

| **#** | **Search Terms** | **Hits** |
| --- | --- | --- |
| #1 | TS = "Neoplasm, Urinary Bladder" OR "Urinary Bladder Neoplasm" OR "Bladder Tumors" OR "Bladder Tumor" OR "Tumor, Bladder" OR "Tumors, Bladder" OR "Neoplasms, Bladder" OR "Bladder Neoplasms" OR "Bladder Neoplasm" OR "Neoplasm, Bladder" OR "Urinary Bladder Cancer" OR "Cancer, Urinary Bladder" OR "Malignant Tumor of Urinary Bladder" OR "Cancer of the Bladder" OR "Bladder Cancer" OR "Bladder Cancers" OR "Cancer, Bladder" OR "Cancer of Bladder" | 139,182 |
| #2 | TS = " Blood Transfusion " OR " Blood Transfusions " OR " Transfusion, Blood " OR " Transfusions, Blood " | 132,443 |
| **#3** | **#1 AND #2** | **580** |
