# Supplementary material for: The effect of different timing of blood transfusion on oncological outcomes of patients undergoing radical cystectomy for bladder cancer: a systematic review and meta-analysis
Source: Front Oncol. 2023 Aug 30;13:1223592. doi: 10.3389/fonc.2023.1223592 (PMC10499617; doi:10.3389/fonc.2023.1223592)
Supplement: Supplementary file 3 [file Table_2.docx]

**Table S2:** Characteristics of included studies in this systematic review and meta-analysis

| **Study** | **Year** | **Country** | **Study design** | **Recruitment period** | **Follow-up** | **Patient characteristics** | **Total(n)** | **Blood transfusion** | | **No blood transfusion** | **Type of transfusion** | **Indication to transfuse** | **Baseline characteristics of study patients** | | | | | **pathologic characteristics of study patients** | | | | | **Blood loss and transfusion characteristics of study patients** | | **chemotherapy** | |  |
| --- | --- | --- | --- | --- | --- | --- | --- | --- | --- | --- | --- | --- | --- | --- | --- | --- | --- | --- | --- | --- | --- | --- | --- | --- | --- | --- | --- |
|  |  |  |  |  |  |  |  |  |  |  |  |  | **Age (years), mean (s.d.) or median (IQR)** | | **male patients (%)** | **BMI** | **Preoperative hemoglobin level, g/dl, median (IQR)** | **pathologic stage (%)** | | **lymph node positive (%)** | **margin positive (%)** | **high-grade tumors (%)** | **estimated blood loss(ml)** | **blood transfused(mL)** |  |  |  |
|  |  |  |  |  |  |  |  |  |  |  |  |  |  |  |  |  |  |  |  |  |  |  |  |  |  |  |  |
| Brian J. Linder et al.[1] | 2013 | USA | retrospective | 1980-2005 | 10.9 years (IQR: 7.9–15.7) | Following institutional review board approval, the authors reviewed the Mayo Clinic Cystectomy Registry and identified patients treated with RC for BCa at their institution between 1980 and 2005. | 2060 | 1279 | | 781 | transfusion of allogenic red blood cells during RC or within the postoperative hospitalization | discretion of physician | (1):69 (63-76) (2):66 (59–72) | | (1):74.0 (2):91.6 | (1) <30:78.9% (2) >30:21.1% | (1)13.5 (12.2–14.6) (2)14.6 (13.7–15.3) | ≤pT1 | (1):31.9; (2):37.9 | (1):15.9 (2):14.3 | (1):2.8 (2):1.8 | NA | (1)900(600-1300) (2)500(350-700) | 2 (2-4) U | NA | |  |
|  |  |  |  |  |  |  |  |  |  |  |  |  |  |  |  |  |  | pT2 | (1):16.0; (2):19.5 |  |  |  |  |  |  |  |  |
|  |  |  |  |  |  |  |  |  |  |  |  |  |  |  |  |  |  | pT3/4 | (1):39.9; (2):29.9 |  |  |  |  |  |  |  |  |
| Todd M. Morgan et al.[2] | 2013 | USA | retrospective | 2000-2008 | 25.0(11.7-46.5) months | patients who underwent RC at Vanderbilt University Medical Center (VUMC) from 2000 to 2008. | 777 | 323 | | 454 | transfusion of packed red blood cells (PRBCs) either intraoperatively or up until the time of discharge from the hospitalization immediately following RC | discretion of physician | (1):71.9 (2):67.4 | | (1):69.0 (2):85.0 | NA | NA | Ta/T1/Tis/T0 | (1):23.0; (2):39.0 | (1):35.0 (2):27.0 | (1):15.0 (2):8.0 | NA | NA | 2 | (1)3.0% (2)2.0% | |  |
|  |  |  |  |  |  |  |  |  |  |  |  |  |  |  |  |  |  | T2 | (1):26.0; (2):24.0 |  |  |  |  |  |  |  |  |
|  |  |  |  |  |  |  |  |  |  |  |  |  |  |  |  |  |  | T3 | (1):30.0; (2):29.0 |  |  |  |  |  |  |  |  |
|  |  |  |  |  |  |  |  |  |  |  |  |  |  |  |  |  |  | T4 | (1):21.0; (2):7.0 |  |  |  |  |  |  |  |  |
|  |  |  |  |  |  |  |  |  |  |  |  |  |  |  |  |  |  |  |  |  |  |  |  |  |  |  |  |
|  |  |  |  |  |  |  |  |  |  |  |  |  |  |  |  |  |  |  |  |  |  |  |  |  |  |  |  |
| E. Jason Abel et al.[3] | 2014 | USA | retrospective (primary cohort) | 2003-2012 | 18.7(8.1-50.4) months | patients with bladder cancer treated with RC without perioperative chemotherapy between January 1, 2003, and December 31, 2012, at the University of Wisconsin (UW) hospital | 360 | 241 | | 119 | receipt of packed red blood cells either during surgery (intraoperative BT) or during the postsurgical hospitalization (postoperative BT) | discretion of physician | Intraoperative | 68.5 (59.8–78.6) | 77 | 27.5 (24.8–31.0) | NA | ≤pT1 | (1)16.7(2)19(3)34 | (1):26.6(2):19.0(3):14 | NA | NA | NA | (1)2 (2)4.5 (3)2 | no chemotherapy | |  |
|  |  |  |  |  |  |  |  |  |  |  |  |  | Intraoperative and postoperative | 69.1 (60.7–76.9) | 63 | 27.6 (24.3–30.5) |  | pT2 | (1)25(2)24(3)18(4)32 |  |  |  |  |  |  |  |  |
|  |  |  |  |  |  |  |  |  |  |  |  |  | Postoperative | 68.5 (59.0–74.3) | 80 | 27.2 (25.2–30.9) |  |  |  |  |  |  |  |  |  |  |  |
|  |  |  |  |  |  |  |  |  |  |  |  |  | No transfusion | 66.5 (57.5–73.9) | 92 | 27.7 (25.4–30.4) |  | pT3/4 | (1)58.3(2)57(3)48(4)35 |  |  |  |  |  |  |  |  |
| E. Jason Abel et al.[3] | 2014 | USA | retrospective (validation cohort) | 1980-2005 | 11.0(8.0–15.7) years | patients who underwent RC without perioperative chemotherapy for bladder cancer at the Mayo Clinic between 1980 and 2005 | 1770 | 1100 | | 670 | receipt of packed red blood cells either during surgery (intraoperative BT) or during the postsurgical hospitalization (postoperative BT) | discretion of physician | Intraoperative | 68 (63–76) | 74 | 26.6 (24.2–29.6) | 13.8 (12.4–14.9) | ≤pT1 | (1)49(2)44(3)48(4)56 | (1)13.4 (2)17 (3)9 (4)6 | (1)2 (2)3 (3)3 (4)2 | NA | NA | (1)2(2)5(3)2 | no chemotherapy | |  |
|  |  |  |  |  |  |  |  |  |  |  |  |  | Intraoperative and postoperative | 71 (66–77) | 72 | 26.5 (23.5–29.4) | 13.0 (11.4–14.2) | pT2 | (1)16(2)16(3)18(4)20 |  |  |  |  |  |  |  |  |
|  |  |  |  |  |  |  |  |  |  |  |  |  | Postoperative | 70 (63–75) | 76 | 26.5 (23.8–29.4) | 13.6 (12.7–14.6) |  |  |  |  |  |  |  |  |  |  |
|  |  |  |  |  |  |  |  |  |  |  |  |  |  |  |  |  |  |  |  |  |  |  |  |  |  |  |  |
|  |  |  |  |  |  |  |  |  |  |  |  |  | No transfusion | 67 (60–73) | 92 | 27.5 (25.0–30.8) | 14.6 (13.8–15.3) | pT3/4 | (1)35(2)41(3)34(4)24 |  |  |  |  |  |  |  |  |
|  |  |  |  |  |  |  |  |  |  |  |  |  |  |  |  |  |  |  |  |  |  |  |  |  |  |  |  |
|  |  |  |  |  |  |  |  |  |  |  |  |  |  |  |  |  |  |  |  |  |  |  |  |  |  |  |  |
| M. Gierth et al.[4] | 2014 | Germany | retrospective | 1995-2010 | 70.1 months | patients of a university health center undergoing RC with pelvic lymph node dissection (PLND) for urothelial carcinoma of the bladder (UCB) between March 1995 and April 2010 | 350 | 219 | | 113 | ABT was arranged by transfusing packed red blood cells (PRBC). Differentiation in intraoperative and postoperative blood transfusion (IBT and PBT) was performed, whereas IBT is defined as ABT during the surgical treatment and PBT as time of transfusion up until 10 days after RC | discretion of physician | (1) 68 (62–74) (2) 68 (58–74) | | (1)77 (2)90 | (1) 26.3 (24.1–29.0) (2) 26.8 (24.2–29.3) | Preoperative anemia (m < 13 g/dl, w < 12 g/dl): (1)52% (2)33% | Ta/T1/Tis/T0 | (1)33 (2)38 | (1)34 (2)23 | (1)16.0 (2)8.0 | NA | (1)1000(500–1,400) (2)600 (400–1,000) | NA | NA | |  |
|  |  |  |  |  |  |  |  |  |  |  |  |  |  |  |  |  |  | T2 | (1)14 (2)22 |  |  |  |  |  |  |  |  |
|  |  |  |  |  |  |  |  |  |  |  |  |  |  |  |  |  |  | T3 | (1)33 (2)29 |  |  |  |  |  |  |  |  |
|  |  |  |  |  |  |  |  |  |  |  |  |  |  |  |  |  |  | T4 | (1)20 (2)11 |  |  |  |  |  |  |  |  |
|  |  |  |  |  |  |  |  |  |  |  |  |  |  |  |  |  |  |  |  |  |  |  |  |  |  |  |  |
|  |  |  |  |  |  |  |  |  |  |  |  |  |  |  |  |  |  |  |  |  |  |  |  |  |  |  |  |
| Luis A. Kluth et al.[5] | 2014 | USA | retrospective | 1998-2010 | 36.1 (15, 84) months | patients from six institutions who underwent RC and bilateral PLND for UCB between 1998 and 2010. | 2895 | 1128 | | 1767 | peri-operative blood transfusion (PBT) | discretion of physician | (1)67.3(14) (2)67.0(13) | | (1)80.9 (2)79.6 | NA | NA | ≤ pT1 | (1)27.1 (2)32.5 | (1)29.9 (2)25.0 | (1)6.4 (2)5.0 | (1)95.3 (2)93.2 | NA | NA | (1)75.4 (2)78.2 | |  |
|  |  |  |  |  |  |  |  |  |  |  |  |  |  |  |  |  |  | pT2 | (1)22.3 (2)24.3 |  |  |  |  |  |  |  |  |
|  |  |  |  |  |  |  |  |  |  |  |  |  |  |  |  |  |  | pT3/4 | (1)50.6 (2)43.2 |  |  |  |  |  |  |  |  |
| Joong Sub Lee et al.[6] | 2015 | Korea | retrospective | 1991-2012 | (1)35 months (2)44 months | patients from a single institution who underwent RC with PLND from January 1991 to December 2012 | 432 | 315 | | 117 | transfusion of only allogeneic RBC during RC or within the postoperative hospitalization period | discretion of physician | (1)66 (59–73) (2)65 (58.5–70) | | (1)82.2 (2)96.9 | (1)23.2(21.0–24.8) (2)23.8(21.6–25.7) | (1)12.5(11.2–13.7) (2)13.8(12.9–14.5) | pT0/Ta/T1/T2/CIS | (1)59.7 (2)70.9 | (1)22.9 (2)21.4 | (1)2.9 (2)0.6 | (1)81.6 (2)83.8 | (1)950(600–1350) (2)500(375–700) | 4 (2–6) | (1)14.0 (2)2.6 | |  |
|  |  |  |  |  |  |  |  |  |  |  |  |  |  |  |  |  |  | pT3/T4 | (1)40.3 (2)29.1 |  |  |  |  |  |  |  |  |
| Marco Moschini et al.[7] | 2015 | Italy | retrospective | 1990-2013 | 110 months | consecutive nonmetastatic bladder cancer patients treated with RC at a single tertiary care referral center between January 1990 and August 2013 | 1490 | Intraoperative BT | 322 | 910 | transfusion of allogeneic red blood cells during RC or in the postoperative hospitalization | discretion of physician | Intraoperative BT | 68 (61-75) | 80.7 | 25.3 (23.2-27.5) | 12.4 (11.0-13.8) | pT0-pT2 | (1)42.2 (2)40.4 (3)43.3 (4)44.7 | NA | (1)9.6 (2)9.9 (3)9.3 (4)6.4 | (1)71.7 (2)67.1 (3)68.0 (4)69.8 | (1)1400 (962-1900) (2)1500 (1000-2100) (3)800 (500-1400) (4)800 (600-1100) | NA | neoadjuvant | (1)3.4 (2)3.7 (3)3.1 (4)2.5 |  |
|  |  |  |  |  |  |  |  | Intraoperative and Postoperative BT | 161 |  |  |  | Intraoperative and Postoperative BT | 68 (63-74) | 80.7 | 25.3 (23.5-28.0) | 11.7 (10.4-12.9) | pT3 | (1)37.6 (2)37.9 (3)37.1 (4)36.2 |  |  |  |  |  |  |  |  |
|  |  |  |  |  |  |  |  |  |  |  |  |  |  |  |  |  |  |  |  |  |  |  |  |  | adjuvant | (1)28.6 (2)28.6 (3)28.9 (4)24.0 |  |
|  |  |  |  |  |  |  |  | Postoperative BT | 97 |  |  |  | Postoperative BT | 68 (61-74) | 78.4 | 24.9 (23.9-28.0) | 13.0 (11.4-14.0) | pT4 | (1)20.2 (2)21.7 (3)19.6 (4)19.1 |  |  |  |  |  |  |  |  |
|  |  |  |  |  |  |  |  |  |  |  |  |  | no BT | 68 (61-74) | 85.6 | 25.1 (22.7-28.4) | 13.2 (11.7-14.3) |  |  |  |  |  |  |  |  |  |  |
| Heather J Chalfin et al.[8] | 2016 | USA | retrospective | 2010-2013 | 7.8 months | The Johns Hopkins Radical Cystectomy database was queried for patients who had undergone NAC followed by RC from 2010 to 2013 | NA | NA | | NA | NA | NA | NA | | NA | NA | NA | NA | | NA | NA | NA | NA | NA | NA | |  |
|  |  |  |  |  |  |  |  |  |  |  |  |  |  |  |  |  |  |  |  |  |  |  |  |  |  |  |  |
|  |  |  |  |  |  |  |  |  |  |  |  |  |  |  |  |  |  |  |  |  |  |  |  |  |  |  |  |
| Marco Moschini et al.[9] | 2016 | Italy | retrospective | 1995-2013 | 89 months | patients with nonmetastatic BCa with available ABO type, treated with RC and extended pelvic lymph node dissection at a single tertiary care referral center between January 1995 and August 2013 |  |  | |  | intraoperative blood transfusion | NA | NA | | NA | NA | NA | NA | | NA | NA | NA | NA | NA | NA | |  |
|  |  |  |  |  |  |  |  |  |  |  |  |  |  |  |  |  |  |  |  |  |  |  |  |  |  |  |  |
|  |  |  |  |  |  |  |  |  |  |  |  |  |  |  |  |  |  |  |  |  |  |  |  |  |  |  |  |
|  |  |  |  |  |  |  |  |  |  |  |  |  |  |  |  |  |  |  |  |  |  |  |  |  |  |  |  |
| Alexander Buchner et al.[10] | 2017 | Germany | retrospective | 2004-2014 | 26(12-61) months | petients who underwent radical cystectomy with pelvic lymph-node dissection from April 2004 to December 2014. | 722 | 317 | | 405 | intraoperative and/or postoperative BT | discretion of physician | 70 (62–76) | | 77 | 26.0 (23.7–29.0) | 13.5 (12.0–14.7) | ≤T2 | 55 | 24 | 13 | 89 | (1)1000(600-1600) (2)500(350-800) | 2U | NA | |  |
|  |  |  |  |  |  |  |  |  |  |  |  |  |  |  |  |  |  | T3-4 | 45 |  |  |  |  |  |  |  |  |
| Juan J. Chipollini et al.[11] | 2017 | USA | retrospective | 2008-2015 | 27.5(8.3-40.5) months | 1026 consecutive patients who underwent RC from January2008 to January 2015 after excluding those with nonurothelial carcinoma etiology or with missing transfusion data and follow-up | 1026 | 341 | | 685 | transfusion of leukoreduced packed red blood cells intraoperatively or within the postoperative hospitalization period | discretion of physician | (1)69.3 (60.5-76.6) (2)70 (62.3-76.9) | | (1)66.8 (2)81.5 | (1)28.4 (5.9) (2)28.3 (6.2) | (1)11.4 (13.6) (2)13.6 (1.5) | ≤pT1 | (1)40.5 (2)40.5 | (1)23.2 (2)19.6 | NA | NA | (1)1237.6 (757.1) (2)619.1 (447.3) | 2 (2-4) U | (1)67.1 (2)23 | |  |
|  |  |  |  |  |  |  |  |  |  |  |  |  |  |  |  |  |  | pT2 | (1)16.7 (2)21.2 |  |  |  |  |  |  |  |  |
|  |  |  |  |  |  |  |  |  |  |  |  |  |  |  |  |  |  | pT3 | (1)26.7 (2)25.9 |  |  |  |  |  |  |  |  |
|  |  |  |  |  |  |  |  |  |  |  |  |  |  |  |  |  |  | pT4 | (1)16.1 (2)12.3 |  |  |  |  |  |  |  |  |
| Siemens, D. R. et al.[12] | 2017 | Canada | retrospective | 2000-2008 | NA | patients that underwent RC in Ontario for UBC after excluding those patients receiving neo-adjuvant chemotherapy and/or preoperative radiotherapy | 2593 | 1608 | | 985 | allogeneic red blood cell transfusion | NA | NA | | (1)68 (2)87 | NA | NA | <T2 | (1)17 (2)22 | (1)28 (2)24 | NA | NA | NA | NA | (1)16 (2)17 | |  |
|  |  |  |  |  |  |  |  |  |  |  |  |  |  |  |  |  |  | T2 | (1)20 (2)25 |  |  |  |  |  |  |  |  |
|  |  |  |  |  |  |  |  |  |  |  |  |  |  |  |  |  |  | T3 | (1)38 (2)37 |  |  |  |  |  |  |  |  |
|  |  |  |  |  |  |  |  |  |  |  |  |  |  |  |  |  |  | T4 | (1)26 (2)16 |  |  |  |  |  |  |  |  |
| Sumeet Syan-Bhanvadia et al.[13] | 2017 | USA | retrospective | 2010-2014 | 3.1 (0-5.1) years | From April 2010 to June 2014, 223 consecutive patients underwent RC by a single surgeon employing a restrictive approach to PBT. Of these, 173 patients met inclusion criteria | 173 | 46 | | 127 | allogeneic blood cell transfusion during RC or hospital-stay | NA | 70 (38–93) | | (1)63 (2)85 | ≥25: (1)71.4 (2)48.8 | NA | ≥pT3 (1)52.5 (2)20.5 | | (1)15.2 (2)5.5 | NA | NA | (1)500 (75–1,800) (2)350 (100–1,600) | 2 (1–12) U | neoadjuvant | (1)50.0 (2)28.4 |  |
|  |  |  |  |  |  |  |  |  |  |  |  |  |  |  |  |  |  |  |  |  |  |  |  |  |  |  |  |
|  |  |  |  |  |  |  |  |  |  |  |  |  |  |  |  |  |  |  |  |  |  |  |  |  | adjuvant | (1)10.9 (2)9.5 |  |
|  |  |  |  |  |  |  |  |  |  |  |  |  |  |  |  |  |  |  |  |  |  |  |  |  |  |  |  |
| Marc A. Furrer et al.[14] | 2018 | Switzerland | retrospective | 2000-2015 | 39 (15–92) months | 1025 consecutive patients treated at the Department of Urology, University Hospital Bern, between 1 January 2000 and 31 December 2015. We excluded patients who underwent RC for nonurothelial carcinoma (n = 104) and those with insufficient follow-up,resulting in the inclusion of 885 patients | 885 | PRBC + FFP transfusion | 80 | 618 | perioperative administration of packed red blood cell (PRBC) and fresh frozen plasma (FFP) | NA | PRBC + FFP transfusion | 71 (64–77) | 75 | 25 (23–30) | 11.7 (9.9–13.1) | ≤pT1 | (1)21.3 (2)27.3 (3)31.8 | (1)25.1 (2)33.1 (3)25 | NA | NA | NA | NA | (1)26.3 (2)18.2 (3)13.3 | |  |
|  |  |  |  |  |  |  |  |  |  |  |  |  | PRBC transfusion | 72 (65–78) | 68.4 | 26 (23–28) | 12.3 (10.9–13.6) | pT2 | (1)23.8 (2)23.5 (3)29.1 |  |  |  |  |  |  |  |  |
|  |  |  |  |  |  |  |  | PRBC transfusion | 187 |  |  |  | No transfusion | 67.1 (59.4–74.3) | 76.2 | 26 (23–29) | 13.6 (12.5–14.6) | pT3 | (1)32.5 (2)36.9 (3)32.8 |  |  |  |  |  |  |  |  |
|  |  |  |  |  |  |  |  |  |  |  |  |  |  |  |  |  |  | pT4 | (1)22.5 (2)12.3 (3)6.1 |  |  |  |  |  |  |  |  |
| Malte W. Vetterlein et al.[15] | 2018 | Germany | retrospective | 2011.1-2011.12 | 26 (21–30) months | 679 men and women undergoing RC for high-grade non-muscle-invasive or muscle-invasive bladder cancer at 18 tertiary care centres in Germany, Austria and Italy between 1 January 2011 and 31 December 2011 | 611 | 315 | | 296 | any transfusion of allogeneic red blood cells during RC or within the postoperative inpatient stay | discretion of physician | (1)69.2 (10.2) (2)67.0 (9.7) | | (1)72.4 (2)88.9 | (1)26.4 (4.8) (2)27.3 (4.6) | NA | ≤pT2 | (1)48.9 (2)60.5 | (1)27.9 (2)23.7 | (1)9.5 (2)5.7 | NA | (1)1000 (600–1500) (2)500 (400–800) | NA | neoadjuvant | (1)2.2 (2)1.7 |  |
|  |  |  |  |  |  |  |  |  |  |  |  |  |  |  |  |  |  |  |  |  |  |  |  |  |  |  |  |
|  |  |  |  |  |  |  |  |  |  |  |  |  |  |  |  |  |  | ≥pT3 | (1)51.1 (2)39.5 |  |  |  |  |  | adjuvant | (1)17.5 (2)18.2 |  |
|  |  |  |  |  |  |  |  |  |  |  |  |  |  |  |  |  |  |  |  |  |  |  |  |  |  |  |  |
| Leonidas N. Diamantopoulos et al.[16] | 2021 | USA | retrospective | 2000-2018 | 61.5 months | consecutive patients who underwent RC between 2000 and 2018 for histologically confirmed bladder cancer (any histology). Patients with aborted RC, RC for other causes, or partial cystectomy were excluded | 747 | 238 | | 509 | PBT was defined as red blood cell transfusion during RC (intraoperative) or postoperative hospitalization | NA | (1)68 (60-75) (2)67 (59-74) | | (1)71 (2)81 | NA | NA | <pT2 | (1)40 (2)49 | (1)12 (2)8 | (1)14 (2)9 | NA | (1)1000 (600-1700) (2)400 (200-600) | NA | NA | |  |
|  |  |  |  |  |  |  |  |  |  |  |  |  |  |  |  |  |  | pT2 | (1)14 (2)16 |  |  |  |  |  |  |  |  |
|  |  |  |  |  |  |  |  |  |  |  |  |  |  |  |  |  |  | pT3/T4 | (1)47 (2)35 |  |  |  |  |  |  |  |  |
| Todd M. Morgan et al.[17] | 2010 | NA | NA | 2000-2007 | 16.1 (1-91.5) months | 789 consecutive patients undergoing RC from 2000-2007 for urothelial carcinoma of the bladder with or without other histologic elements | 789 | 321 | | 468 | transfusion of packed red blood cells during cystectomy or within the postoperative hospitalization | NA | NA | | NA | NA | NA | NA | | NA | NA | NA | NA | NA | NA | |  |
|  |  |  |  |  |  |  |  |  |  |  |  |  |  |  |  |  |  |  |  |  |  |  |  |  |  |  |  |
|  |  |  |  |  |  |  |  |  |  |  |  |  |  |  |  |  |  |  |  |  |  |  |  |  |  |  |  |
|  |  |  |  |  |  |  |  |  |  |  |  |  |  |  |  |  |  |  |  |  |  |  |  |  |  |  |  |
| Gild P et al.[18] | 2017 | NA | NA | 2011 | 26 months (IQR: 21-30 months | 679 patients undergoing RC at 18 European tertiary care centers in 2011. Patients with missing information on PBT, ASA physical status, follow-up, survival data, or disease recurrence. | 525 | 275 | | 250 | NA | NA | NA | | NA | NA | NA | NA | | NA | NA | NA | (1)1000(650-1600) (2) 570(400-800) | NA | NA | |  |
|  |  |  |  |  |  |  |  |  |  |  |  |  |  |  |  |  |  |  |  |  |  |  |  |  |  |  |  |
|  |  |  |  |  |  |  |  |  |  |  |  |  |  |  |  |  |  |  |  |  |  |  |  |  |  |  |  |
|  |  |  |  |  |  |  |  |  |  |  |  |  |  |  |  |  |  |  |  |  |  |  |  |  |  |  |  |
| Diamantopoulos L.N. et al.[19] | 2020 | USA | NA | NA | 66 (60 -72) months | Pts with BC treated with RC were retrospectively identified | 784 | 238 | | 546 | red blood cell transfusion during RC or postoperative hospitalization | NA | NA | | NA | NA | NA | NA | | NA | NA | NA | NA | NA | NA | |  |
|  |  |  |  |  |  |  |  |  |  |  |  |  |  |  |  |  |  |  |  |  |  |  |  |  |  |  |  |
| Ayman Soubra et al.[20] | 2015 | USA | retrospective | 1992-2009 | 21 (8-52) months | Using the Surveillance, Epidemiology, and End Results-Medicare data set from 1992-2009, we identified 5462 patients with bladder cancer,who underwent RC as primary therapy | 5462 | 1139 | | 4323 | PRBC | NA | 74 (70–78) | | NA | NA | NA | NA | NA | NA | NA | NA | NA | NA | NA | |  |
| Yuh et al.[21] | 2014 | USA | retrospective | 2003-2011 | 52 months | A total of 241 consecutive patients underwent RARC, extended pelvic lymph node dissection, and urinary diversion between 2003 and 2011. | 162 | NA | | NA | NA | NA | 70 (62–78) | | NA | NA | NA | NA | NA | NA | NA | NA | 400 | NA | NA | |  |
| Sadeghi et al.[22] | 2012 | USA | retrospective | 1989-2010 | 25.5 (1–164) months | The Columbia University Urologic Oncology Database was reviewed for patients who underwent a RC from 1989 to 2010 (n = 638). | 638 | 209 | | 429 | PRBC | discretion of Physician | 67.6 (11.1) | | NA | NA | NA | NA | NA | NA | 9.1 | NA | NA | NA | NA | |  |
| Julien et al.[23] | 2023 | Spain | retrospective | 2015-2022 | 23 months | Patients treated for UCB in 15 academic institutions. | 635 | 95 | | 540 | during surgery (intraoperative blood transfusions,  iBT) or during the first 30 days after surgery (post-operative blood transfusions, pBT) | discretion of Physician | 67 (60-73) | | 84.7 | 25.8 (23.7-28.4) | NA | ≤pT1  pT2  pT3  pT4 | 54.2  15.4  24.1  6.3 | NA | 4.7 | NA | 250 (199-460) | NA | 49.1 | |  |

Abbreviations: BMI, body mass index; IQR, inter quartile range; SD, standard deviation; USA, United States of America; PRBC, packed red blood cell; RC, radical cystectomy; BCa, bladder cancer; PBT, perioperative blood transfusion; BT, blood transfusion; PLND, pelvic lymph node dissection; UCB, urothelial carcinoma of the bladder; ABT, allogenic blood transfusion; (1) patients receiving blood transfusion and (2) patients without blood transfusion.

1. Linder, B.J., et al., *The impact of perioperative blood transfusion on cancer recurrence and survival following radical cystectomy.* Eur Urol, 2013. **63**(5): p. 839-45.

2. Morgan, T.M., et al., *The relationship between perioperative blood transfusion and overall mortality in patients undergoing radical cystectomy for bladder cancer.* Urol Oncol, 2013. **31**(6): p. 871-7.

3. Abel, E.J., et al., *Perioperative blood transfusion and radical cystectomy: does timing of transfusion affect bladder cancer mortality?* Eur Urol, 2014. **66**(6): p. 1139-47.

4. Gierth, M., et al., *The effect of intra- and postoperative allogenic blood transfusion on patients' survival undergoing radical cystectomy for urothelial carcinoma of the bladder.* World J Urol, 2014. **32**(6): p. 1447-53.

5. Kluth, L.A., et al., *Impact of peri-operative blood transfusion on the outcomes of patients undergoing radical cystectomy for urothelial carcinoma of the bladder.* BJU Int, 2014. **113**(3): p. 393-8.

6. Lee, J.S., et al., *The prognostic impact of perioperative blood transfusion on survival in patients with bladder urothelial carcinoma treated with radical cystectomy.* Korean J Urol, 2015. **56**(4): p. 295-304.

7. Moschini, M., et al., *Effect of allogeneic intraoperative blood transfusion on survival in patients treated with radical cystectomy for nonmetastatic bladder cancer: Results from a single high-volume institution.* Clinical Genitourinary Cancer, 2015. **13(6)**: p. 562-567.

8. Chalfin, H.J., et al., *Blood Transfusion is Associated with Increased Perioperative Morbidity and Adverse Oncologic Outcomes in Bladder Cancer Patients Receiving Neoadjuvant Chemotherapy and Radical Cystectomy.* Ann Surg Oncol, 2016. **23**(8): p. 2715-22.

9. Moschini, M., et al., *Timing of blood transfusion and not ABO blood type is associated with survival in patients treated with radical cystectomy for nonmetastatic bladder cancer: Results from a single high-volume institution.* Urol Oncol, 2016. **34**(6): p. 256.e7-256.e13.

10. Buchner, A., et al., *Dramatic impact of blood transfusion on cancer-specific survival after radical cystectomy irrespective of tumor stage.* Scand J Urol, 2017. **51**(2): p. 130-136.

11. Chipollini, J.J., et al., *Perioperative Transfusion of Leukocyte-depleted Blood Products in Contemporary Radical Cystectomy Cohort Does Not Adversely Impact Short-term Survival.* Urology, 2017. **103**: p. 142-148.

12. Siemens, D.R., et al., *Peri-operative allogeneic blood transfusion and outcomes after radical cystectomy: a population-based study.* World J Urol, 2017. **35**(9): p. 1435-1442.

13. Syan-Bhanvadia, S., et al., *Restrictive transfusion in radical cystectomy is safe.* Urol Oncol, 2017. **35**(8): p. 528.e15-528.e21.

14. Furrer, M.A., et al., *Impact of Packed Red Blood Cells and Fresh Frozen Plasma Given During Radical Cystectomy and Urinary Diversion on Cancer-related Outcome and Survival: An Observational Cohort Study.* Eur Urol Focus, 2018. **4**(6): p. 916-923.

15. Vetterlein, M.W., et al., *Peri-operative allogeneic blood transfusion does not adversely affect oncological outcomes after radical cystectomy for urinary bladder cancer: a propensity score-weighted European multicentre study.* BJU Int, 2018. **121**(1): p. 101-110.

16. Diamantopoulos, L.N., et al., *Patterns and timing of perioperative blood transfusion and association with outcomes after radical cystectomy.* Urol Oncol, 2021. **39**(8): p. 496.e1-496.e8.

17. Morgan, T.M., et al., *Perioperative blood transfusion increases the risk of overall mortality in patients undergoing radical cystectomy for bladder cancer.* Journal of Urology, 2010. **1)**: p. e705-e706.

18. Gild, P., et al., *Perioperative allogeneic blood transfusion does not adversely impact survival after radical cystectomy for urinary bladder cancer-a competingrisks analysis from a multi-institutional European series.* European Urology, Supplements, 2017. **16(3)**: p. e1578-e1579.

19. Diamantopoulos, L.N., et al., *Perioperative blood transfusion and postoperative outcomes in patients undergoing radical cystectomy for bladder cancer.* Journal of Clinical Oncology. Conference, 2020. **38**(15).

20. Soubra, A., et al., *Effect of perioperative blood transfusion on mortality for major urologic malignancies.* Clin Genitourin Cancer, 2015. **13**(3): p. e173-81.

21. Yuh, B., et al., *Intermediate-term oncologic outcomes of robot-assisted radical cystectomy for urothelial carcinoma.* J Endourol, 2014. **28**(8): p. 939-45.

22. Sadeghi, N., et al., *The impact of perioperative blood transfusion on survival following radical cystectomy for urothelial carcinoma.* Can J Urol, 2012. **19**(5): p. 6443-9.

23. Sarkis, J., et al., *Do perioperative blood transfusions impact oncological outcomes of robot-assisted radical cystectomy with intracorporeal urinary diversion? Results from a large multi-institutional registry.* Minerva Urology and Nephrology, 2023. **75**(1): p. 50-58.
