# Supplementary material for: The effect of different timing of blood transfusion on oncological outcomes of patients undergoing radical cystectomy for bladder cancer: a systematic review and meta-analysis
Source: Front Oncol. 2023 Aug 30;13:1223592. doi: 10.3389/fonc.2023.1223592 (PMC10499617; doi:10.3389/fonc.2023.1223592)
Supplement: Supplementary file 4 [file Table_3.docx]

**Table S3. Newcastle-Ottawa Scale for assessing the quality of studies in meta-analysis**

| **Study** | **Selection** | | | | **Comparability** | **Outcomes** | | | **Scores** |
| --- | --- | --- | --- | --- | --- | --- | --- | --- | --- |
|  | **Representativeness of Exposed Cohort** | **Selection of the Non-Exposed Cohort** | **Ascertainment of Exposure** | **Demonstration That Outcome of Interest Was Not Present at Start of Study** | **Comparability of Cohorts on the Basis of the Design or Analysis** | **Assesement of Outcome** | **Was Follow-Up Long enough for Outcomes to Occur** | **Adequacy of Follow Up Cohorts** |  |
| Brian J. Linder et al. 2013 |  | ⭐ | ⭐ | ⭐ | ⭐⭐ | ⭐ | ⭐ | ⭐ | 8 |
| Todd M. Morgan et al. 2013 |  | ⭐ | ⭐ | ⭐ | ⭐ | ⭐ | ⭐ | ⭐ | 7 |
| E. Jason Abel et al. (primary cohort) 2014 |  | ⭐ | ⭐ | ⭐ | ⭐ | ⭐ | ⭐ | ⭐ | 8 |
| E. Jason Abel et al. (validation cohort) 2014 |  | ⭐ | ⭐ | ⭐ | ⭐⭐ | ⭐ | ⭐ | ⭐ | 8 |
| M. Gierth et al. 2014 |  | ⭐ | ⭐ | ⭐ | ⭐⭐ | ⭐ | ⭐ | ⭐ | 8 |
| Luis A. Kluth et al. 2014 |  | ⭐ | ⭐ | ⭐ | ⭐⭐ | ⭐ | ⭐ | ⭐ | 8 |
| Joong Sub Lee et al. 2015 |  | ⭐ | ⭐ | ⭐ | ⭐⭐ | ⭐ | ⭐ | ⭐ | 8 |
| Marco Moschini et al. 2015 |  | ⭐ | ⭐ | ⭐ | ⭐⭐ | ⭐ | ⭐ | ⭐ | 8 |
| Heather J Chalfin et al. 2016 |  | ⭐ | ⭐ | ⭐ | ⭐ | ⭐ | ⭐ | ⭐ | 7 |
| Marco Moschini et al. 2016 |  | ⭐ | ⭐ | ⭐ | ⭐⭐ | ⭐ | ⭐ | ⭐ | 8 |
| Alexander Buchner et al. 2017 |  | ⭐ | ⭐ | ⭐ | ⭐⭐ | ⭐ | ⭐ | ⭐ | 8 |
| Juan J. Chipollini et al. 2017 |  | ⭐ | ⭐ | ⭐ | ⭐⭐ | ⭐ | ⭐ | ⭐ | 8 |
| Siemens, D. R. et al. 2017 | ⭐ | ⭐ | ⭐ | ⭐ | ⭐⭐ | ⭐ | ⭐ | ⭐ | 9 |
| Sumeet Syan-Bhanvadia et al. 2017 |  | ⭐ | ⭐ | ⭐ | ⭐⭐ | ⭐ | ⭐ | ⭐ | 8 |
| Marc A. Furrer et al. 2018 |  | ⭐ | ⭐ | ⭐ | ⭐⭐ | ⭐ | ⭐ | ⭐ | 8 |
| Malte W. Vetterlein et al. 2018 |  | ⭐ | ⭐ | ⭐ | ⭐⭐ | ⭐ | ⭐ | ⭐ | 8 |
| Leonidas N. et al. 2021 |  |  | ⭐ | ⭐ | ⭐⭐ | ⭐ | ⭐ | ⭐ | 7 |
| Ayman Soubra et al. 2015 |  | ⭐ | ⭐ | ⭐ | ⭐ | ⭐ | ⭐ | ⭐ | 7 |
| Yuh et al. 2014 |  | ⭐ | ⭐ | ⭐ | ⭐ | ⭐ | ⭐ | ⭐ | 7 |
| Sadeghi et al. 2012 |  | ⭐ | ⭐ | ⭐ | ⭐ | ⭐ | ⭐ | ⭐ | 7 |
| Julien et al. 2023 |  | ⭐ | ⭐ | ⭐ | ⭐⭐ | ⭐ | ⭐ | ⭐ | 8 |

**Note: A study can be awarded a maximum of one star for each numbered item within the Selection and Outcome categories. A maximum of two stars can be given for Comparability.**
