# Supplementary material for: The effect of different timing of blood transfusion on oncological outcomes of patients undergoing radical cystectomy for bladder cancer: a systematic review and meta-analysis
Source: Front Oncol. 2023 Aug 30;13:1223592. doi: 10.3389/fonc.2023.1223592 (PMC10499617; doi:10.3389/fonc.2023.1223592)
Supplement: Supplementary file 5 [file Table_4.docx]

**Table S4 Univariable meta regression analysis of all-cause mortality**

| **Variables** | **B** | **SE** | **P value** |
| --- | --- | --- | --- |
| **Year** | 0.0127 | 0.0154 | 0.4108 |
| **Follow-up** | 0.0007 | 0.0010 | 0.4817 |
| **Age** | -0.0014 | 0.0331 | 0.9652 |
| **BMI** | -0.0818 | 0.0574 | 0.1536 |
| **Hb** | -0.0541 | 0.0735 | 0.4616 |
| **EBL** | 0.0000 | 0.0002 | 0.9329 |
| **Chemotherapy** | -0.0037 | 0.0020 | 0.0661 |
| **Stage2** | -0.0029 | 0.0059 | 0.6309 |
| **LN** | 0.0046 | 0.0061 | 0.4539 |
| **Margin positive** | 0.0171 | 0.0098 | 0.0799 |
| **High grade tumor** | -0.0062 | 0.0031 | 0.0457 |
| **Sex** | -0.0054 | 0.0097 | 0.5775 |

**B, regression coefficient; SE, standard error; BMI, body mass index; Hb, hemoglobin level; EBL, estimated blood loss; Chemotherapy, percentage of patients receiving chemotherapy; Stage 2, percentage of patients with pathological stage greater than T2; LN, percentage of patients with positive lymph nodes; Margin positive, percentage of patients with positive margin; High grade tumor, percentage of patients with high grade tumor; Sex, percentage of male patients.**
