# Supplementary material for: The effect of different timing of blood transfusion on oncological outcomes of patients undergoing radical cystectomy for bladder cancer: a systematic review and meta-analysis
Source: Front Oncol. 2023 Aug 30;13:1223592. doi: 10.3389/fonc.2023.1223592 (PMC10499617; doi:10.3389/fonc.2023.1223592)
Supplement: Supplementary file 6 [file Table_5.docx]

**Table S5 Univariable meta regression analysis of cancer-specific mortality**

| **Variables** | **B** | **SE** | **P value** |
| --- | --- | --- | --- |
| **Year** | 0.0038 | 0.0203 | 0.8510 |
| **Follow-up** | 0.0011 | 0.0009 | 0.2311 |
| **Age** | 0.0010 | 0.0399 | 0.9799 |
| **BMI** | -0.1149 | 0.0846 | 0.1743 |
| **Hb** | -0.0942 | 0.0922 | 0.3067 |
| **EBL** | 0.0001 | 0.0002 | 0.7267 |
| **Chemotherapy** | -0.0046 | 0.0015 | 0.0027 |
| **Stage2** | -0.0092 | 0.0055 | 0.0954 |
| **LN** | 0.0060 | 0.0085 | 0.4819 |
| **Margin positive** | -0.0096 | 0.0111 | 0.3876 |
| **High grade tumor** | -0.0100 | 0.0039 | 0.0099 |
| **Sex** | -0.0073 | 0.0109 | 0.5055 |

**B, regression coefficient; SE, standard error; BMI, body mass index; Hb, hemoglobin level; EBL, estimated blood loss; Chemotherapy, percentage of patients receiving chemotherapy; Stage 2, percentage of patients with pathological stage greater than T2; LN, percentage of patients with positive lymph nodes; Margin positive, percentage of patients with positive margin; High grade tumor, percentage of patients with high grade tumor; Sex, percentage of male patients.**
