# Supplementary material for: The effect of different timing of blood transfusion on oncological outcomes of patients undergoing radical cystectomy for bladder cancer: a systematic review and meta-analysis
Source: Front Oncol. 2023 Aug 30;13:1223592. doi: 10.3389/fonc.2023.1223592 (PMC10499617; doi:10.3389/fonc.2023.1223592)
Supplement: Supplementary file 7 [file Table_6.docx]

**Table S6. Univariable meta regression analysis of disease recurrence**

| **Variables** | **B** | **SE** | **P value** |
| --- | --- | --- | --- |
| **Year** | 0.0309 | 0.0136 | 0.0226 |
| **Follow-up** | -0.0003 | 0.0011 | 0.8165 |
| **Age** | -0.0109 | 0.0396 | 0.7830 |
| **BMI** | -0.0669 | 0.0992 | 0.5001 |
| **Hb** | -0.0772 | 0.1335 | 0.5631 |
| **EBL** | -0.0001 | 0.0002 | 0.4829 |
| **Chemotherapy** | -0.0029 | 0.0021 | 0.1645 |
| **Stage2** | -0.0043 | 0.0060 | 0.4708 |
| **LN** | 0.0013 | 0.0076 | 0.8665 |
| **Margin positive** | 0.0124 | 0.0151 | 0.4126 |
| **High grade tumor** | -0.0032 | 0.0037 | 0.3991 |
| **Sex** | -0.0124 | 0.0092 | 0.1775 |

**B, regression coefficient; SE, standard error; Year, year of publication; Follow-up, length of follow-up period; BMI, body mass index; Hb, hemoglobin level; EBL, estimated blood loss; Chemotherapy, percentage of patients receiving chemotherapy; Stage 2, percentage of patients with pathological stage greater than T2; LN, percentage of patients with positive lymph nodes; Margin positive, percentage of patients with positive margin; High grade tumor, percentage of patients with high grade tumor; Sex, percentage of male patients.**
